# The potential use of mass timber in mid-to high-rise construction and the associated carbon benefits in the United States

**DOI:** 10.1371/journal.pone.0298379

**Published:** 2024-03-20

**Authors:** Prakash Nepal, Jeffrey P. Prestemon, Indroneil Ganguly, Vaibhav Kumar, Richard D. Bergman, Neelam C. Poudyal

**Affiliations:** 1 Forest Products Laboratory, USDA Forest Service, Madison, Wisconsin, United States of America; 2 Southern Research Station, USDA Forest Service, Research Triangle Park, North Carolina, United States of America; 3 College of the Environment, University of Washington, Seattle, Washington, United States of America; 4 School of Natural Resources, University of Tennessee, Knoxville, Tennessee, United States of America; University of Georgia, UNITED STATES

## Abstract

Nonresidential and mid- to high-rise multifamily residential structures in the United States currently use little wood per unit floor area installed, because earlier building codes lacked provisions for structural wood use in those types of buildings. However, revisions to the International Building Code allow for increased wood use in the form of mass timber, as structural and fire safety concerns have been addressed through new science-based design standards and through newly specified construction materials and measures. This study used multiple models to describe alternative futures for new construction, mass timber adoption rates, and the associated carbon benefits in higher than three-story buildings in the United States. The use of mass timber, in place of traditional constructions (i.e., structures dominated by concrete and steel), in projected new higher than three-story buildings was shown to provide combined carbon benefits (i.e., global warming mitigation benefits), including avoided embodied carbon emissions due to the substitution of non-wood alternatives and additional biogenic carbon storage in mass timber materials, of between 9.9 and 16.5 million t CO_2_e/yr spanning 50 years, 2020 to 2070. These carbon benefits equate to 12% to 20% of the total U.S. harvested wood products carbon storage for 2020. Future research is needed to understand how greater mass timber adoption leads to changes in forest product markets, land use, and total forest sector carbon.

## Introduction

The majority of single-family and low-rise multifamily residential housing units in the United States is built primarily of wood, with little opportunity for increased wood use in the structural components in those building types. In contrast, mid- to high-rise mixed-use buildings and all nonresidential buildings in the United States have historically used relatively little wood, favoring fossil fuel-intensive building materials such as concrete and steel. Lack of wood demand for these generally larger structures is due in part to the historical rules contained in the International Building Codes (IBCs). Until recently, the IBC did not have provisions for the use of structural wood components in buildings exceeding certain heights and square footage. In 2021, these standards were updated to allow for the use of mass timber in both taller and more expansive structures [1, 2]. The 2021 IBC describes codes for new construction types (IV-A, IV-B, IV-C) that allow for these products; for IV-A building types, the maximum allowable number of stories above grade plane is 18 stories with maximum allowable floor area of 184,500 square feet (17,140 square meters) [2, 3]. These changes open up new opportunities for the use of structural members made from wood (collectively referred to as mass timber), including cross-laminated timber (CLT), nail-laminated timber (NLT), glue-laminated beams (glulam), and laminated veneer lumber (LVL) [4]. Nevertheless, the future of mass timber in nonresidential and high-rise multifamily residential structures in the United States is currently constrained by many factors, including a lack of knowledge among architects, builders, contractors, and insurers regarding design, permitting, and building [5–7]. Mass timber may be considered cost-prohibitive when compared to concrete building systems and requires additions of other building products to make it acceptable in many applications [8–10]. Other concerns include that mass timber has restrictive spans and that it can absorb moisture in some cases [11].

Despite these barriers, the number of mass timber projects in the United States is growing, aided by continuous scientific research efforts demonstrating the structural, aesthetic, acoustic, seismic, and fire performance advantages of mass timber structures and bolstered by the efforts of non-profit organizations and CLT manufacturers to raise awareness of mass timber benefits [9, 12–15]. For example, as of December, 2022, 1,677 projects involving modern mass timber and post-and-beam use in multifamily, commercial, and institutional structures were recorded across all 50 states in the United States, among which 767 were already completed or under construction, and the remaining 910 were in the design phase [16]. To put this into perspective, the number of mass timber projects recorded in December, 2022, represented an increase of more than 114% over the number of active projects recorded in March, 2020 [17]. However, note that the current (2022) level of wood consumption for mass timber in North America (0.362 million m^3^ [18]) still constitutes a small fraction (about 0.4%) of total softwood lumber consumption in the region [19].

Literature on how markets for mass timber products could be expanded under various scenarios is limited for several reasons. First, a lack of econometric models of nonresidential construction has been a technical barrier to developing credible scenarios of future mass timber demand. Second, nonresidential construction data are only reported periodically and at uneven intervals. In addition, reported data are highly aggregated spatially (to U.S. Census Bureau multi-state Regions), reported at low frequency, and extend back only to the late 1970s. Although it is possible that sufficient data are now available to derive initial estimates of reduced-form equations for construction rates, no study has attempted this so far.

Furthermore, projections of mass timber demand by nonresidential construction analysts have been constrained by the lack of estimates of realistic bounds on potential demand expansion. With the advent of the revised IBC and recent studies uncovering barriers to mass timber demand growth, opportunities now exist for basing such scenarios on research findings (e.g., [20]). Scenarios of possible demand expansion could be based on alternative rates of mass timber market penetration, despite the barriers just described. These alternative rates are suggested under the assumption that several actions may be taken to address barriers to adoption. Possible actions could include government interventions, such as (i) monetary incentives for mass timber use; (ii) the use of information clearinghouses on how to build with mass timber, including offering low-cost or free training, locating these at universities, community colleges, or within agencies; (iii) the setting up of model building programs (demonstration initiatives) that showcase the use of mass timber systems in construction; or (iv) the offering of government-subsidized insurance premiums or the underwriting of insurers who provide insurance to builders and owners. Taylor et al. [21] described how these types of government incentives could be justified through carbon pricing.

Expanded use of mass timber in new nonresidential and mid-to high-rise mixed-use buildings can provide additional carbon emission reduction benefits. Such benefits derive not only from biogenic carbon stored in mass timber materials [22, 23], but also through avoided fossil-fuel emissions due to substitution for non-wood alternative materials that emit more in manufacturing and use [15, 21, 24–31]. Estimates of these benefits, especially at aggregate U.S. regional and national scales, are lacking. In the current study, we attempted to fill these gaps in knowledge in many ways. First, we developed and applied statistical models to project floor areas likely to be added in nonresidential and multifamily residential buildings under varying socioeconomic futures provided by the Intergovernmental Panel on Climate Change (IPCC) inspired contrasting shared socioeconomic pathways (SSPs). Second, we combined these projections of floor areas with the new product diffusion models we developed in this study to project potential adoption of mass timber in new buildings under a range of market adoption scenarios. Finally, we combined the projected volumes of mass timber to be adopted in the alternate scenarios to estimate the associated increased biogenic carbon sequestration and reduced embodied carbon benefits.

## Materials and methods

Our study estimated statistical models of new floor areas installed in nonresidential buildings in the United States by four U.S. Census Bureau Regions (Northeast, Midwest, South and West) using cross-sectional time series (panel data) methods (see Appendix A in the S1 File). The estimated models, along with projections of predictor variables, were then utilized to project new nonresidential floor areas built annually in each of four U.S. regions over the next 50 years (2020–2070) under varying socioeconomic futures described by five SSPs [32]. We then used the projections of new floor areas built in multifamily residential units that were based on recently published projections of multifamily residential units under the same SSPs [22]. These individual projections of new floor areas built in nonresidential and multifamily residential buildings were then integrated with a new-product market diffusion model to investigate potential mass timber adoption levels under a range of market adoption scenarios encompassing low, medium, and high adoption to 100% adoption futures. Finally, a life-cycle assessment (LCA) framework was used to understand the likely greenhouse gas (GHG) emissions as a result of the projected mass timber use in those buildings, under different adoption scenarios. These GHG estimates were then used for developing comparative estimates of carbon benefits (i.e., reduced GHG emissions) due to substitution of mass timber instead of concrete structures. Finally, the study developed an estimate of biogenic carbon stored in mass timber buildings while in use during the functional life of the buildings, followed by biogenic carbon storage in landfills after end-of-life demolition of the mass timber buildings. The estimates of the avoided emissions benefits of mass timber substitution were derived from a published whole building LCA (WBLCA) study that compared embodied fossil emissions of representative 8-, 12-, and 18-story mass timber and functionally equivalent reinforced concrete buildings in three U.S. regions covering raw material extraction (module A1), raw and intermediary material transportation (module A2), material production (module A3), finished material transportation (module A4), and construction stages (moduleA5) [28]. Similarly, the estimates of biogenic carbon stored in mass timber materials were modeled using a harvested wood product (HWP) carbon model [22, 33] that tracked carbon added in and discarded from buildings. A description of each model and approach follows.

### Statistical models of nonresidential construction activities

We estimated statistical models of new floor area built in nonresidential buildings, which were functions of various socioeconomic factors, including regional economic output, interest rates, commodity prices, and wages (as measured by weekly earnings) (S1 Table in S1 File). The description of the specified models and the approach and methods used in estimating the model parameters of nonresidential floor areas follows.

#### Structural model of nonresidential floor space built

There is a lack of guiding theory on the factors driving supply and demand of nonresidential construction in the United States. We can, however, generally describe the construction inverse supply function as follows:

$$P_{i,t}=f(Q_{i,t}^{S},W_{i,t},R_{i,t},Z_{i,t})$$ (1)

where *P* is the per-unit rental price (e.g., in dollars per unit of floor space), *Q*^*S*^ indexes an aggregate physical measure (units) of new nonresidential floor space supplied, *W* is the wage paid to construction labor, *R* is the cost of capital, **Z** indexes all other factors influencing the construction decision (e.g., laws and regulations), and *i* and *t* are region and time indicators. Similarly, the construction inverse demand function can be described as follows:

$$P_{i,t}=g(Q_{i,t}^{D},Y_{i,t},X_{i,t})$$ (2)

where *Q*^*D*^ indexes an aggregate physical measure of new nonresidential floor area demanded, *Y* is an aggregate and potentially exogenous index of aggregate income, **X** indexes all other factors influencing demand for nonresidential floor area, *P* is as previously defined, and *i* and *t* index region and time, as in (Eq 1). A reduced-form version can be found by equating (Eq 1) and (Eq 2) and imposing a market-clearing condition of $Q_{i,t}^{S}=Q_{i,t}^{D}=Q_{i,t}$ and solving for *Q*_*i*,*t*_, a reduced-form equation:

$$Q_{i,t}=h(W_{i,t},R_{i,t},Y_{i,t},Z_{i,t},X_{i,t})$$ (3)

Detailed empirical methods applied for estimating Eq 3 and the Monte Carlo bootstrapping approach to projecting nonresidential floor area built are described in Appendix A of the S1 File.

#### Empirical model of nonresidential construction

We specified empirical models of nonresidential new floor area built, versions of Eq 3. We began by identifying fully specified models, with variables representing those shown in Eq 3. These models accounted for the possible joint determination of the prices of construction materials and labor. Models were cross-sectional annual time series panels, with the time series ranging from 1983 to 2020 and the cross-sections identified as Census regions. The estimated models accounted for regions with intercept shifter indicator variables, predicted floor area under different functional forms, and sometimes involved transformations of the dependent variable.

While elaborate models such as those implied by Eq 3 are beneficial for carrying out inference, they are less amenable to being used for out-of-sample projection purposes due to their large data requirements. Therefore, we defined parsimonious versions of Eq 3 that included only a few variables that could be projected: regional gross domestic product (GDP) and the prices of capital and other materials. Model estimates are shown in S2a Table of S1 File for nonresidential floor area built and S2b Table in S1 File for simple models of GDP and the real Prime rate of interest, which are needed for projections (next section). Final models were linear and logarithmically linear for floor area built, the change in the logarithm of real GDP, and the real Prime rate. Details of the model specifications are provided in Appendix A of the S1 File.

#### Projections of new floor areas

The estimated models of the nonresidential floor area built described in the previous section were utilized to provide alternative projections of new nonresidential floor areas added in the United States from 2020 to 2070 under alternate socioeconomic futures represented by SSPs [32]. These SSPs were developed in conjunction with the fifth climate assessment led by the IPCC [34]. The SSPs describe alternative global visions of future population, economy, technology, land use, and energy usage, in addition to other socioeconomic variables [34]. To account for model uncertainty given assumptions of GDP growth by SSP, we applied bootstrapping methods to project nonresidential floor area built and based our projections of floor area by SSP on the median projection for each region (see Appendix A in the S1 File). For multifamily residential buildings, statistical models were available only for new structures built by region [22]. Therefore, to project floor area in new multifamily residential structures, we estimated the linear trends of average historical average floor area per unit of multifamily residential buildings by U.S Census Regions ([35], S3 Table in S1 File), which we multiplied by the median projected structures of multifamily residential units under the same SSPs reported in Prestemon et al. ([22], S4 Table in S1 File).

#### Scenarios of mass-timber adoption

In our mass timber adoption scenarios, we only considered buildings of four stories and higher. We used the current proportion of four-stories-and-higher nonresidential floor area [36] and multifamily residential buildings [37] to allocate projected total floor areas in this building height category. These data suggest that about 19% of total nonresidential building floor areas, and about 21% of total multifamily residential buildings are shared by the four-stories-and-higher building height category currently and in the recent past (S5 Table in S1 File, [36, 37]).

To estimate the alternative trajectories of mass timber uptake in the projected mid- to high-rise nonresidential and multifamily residential floor buildings, we developed four scenarios representing low, medium, high, and 100% market adoption, separated by two building height groups: four- to six-stories and seven-stories-and-higher. While the low adoption scenarios represented greater market and policy constraints in adopting mass timber, the high adoption scenario represented the opposite, with the medium scenario representing an intermediate level of mass timber adoption pathway [38]. Such modeling was conducted for three groups of building heights and types, including two separate models of four-to-six stories for nonresidential and multifamily residential, respectively, and a single model of seven-stories-and-higher for both nonresidential and multifamily residential buildings. This is based on the premise that the market adoption rates would be different for four-to-six stories residential and multifamily residential buildings but similar for both building types above six stories. The projections of the potential market adoption rates in these four scenarios were obtained by utilizing a new-product repeat purchase model ([39], Appendix B, Eqn S2 in S1 File), the parameters of which were optimized to mimic the historically observed market adoption trajectory of oriented-strand board between 1990 and 2010 (at which point its share of the wood-based structural panel market plateaued). The CLT innovation diffusion model is based on a repeat purchase diffusion model for structural wood products within each specific building category. Hence, the parameters of the base model (i.e., the coefficients of trial purchase, repeat purchase, lack of base information, and rate of information dissemination—see Appendix B in S1 File) were altered so as to achieve simulated low, medium, and high market adoption rates for mass timber.

The challenges of estimating product diffusion model parameters of innovative new products are well established in the literature, confirming that these estimated parameters are sensitive to the number of observations available [40]. Often, unrelated literature values or ad hoc estimates are poorly conclusive and are highly dependent on the assumed market potential [41]. Given that CLT as a structural building material is new in the United States, we used the established diffusion parameters of OSB, at the point of its introduction in the United States. OSB was an innovative structural building material displacing plywood as the dominant material for similar applications [39]. Instead of using an ad hoc set of parameters, for this study we used the parameters for OSB diffusion as starting values and used existing CLT adoption data to scale the CLT diffusion model. For long-term estimates, we adjusted the parameters to develop multiple diffusion scenarios.

The 100% adoption scenario represents the potential amounts of mass timber use, after satisfying current building code requirements and under the assumption that there are no market barriers or policy constraints for adopting mass timber. Scenarios of potential mass timber use was based on the CLT and glulam use intensity per square meter of buildings reported by Dolan et al. [42], which indicated a combined CLT and glulam use factors of 0.26, 0.33 and 0.24 m^3^/m^2^ for four to six stories, six to 12 stories, and higher than 13-story building height groups, respectively (S6 Table in S1 File). Somewhat contrary to expectations, the lower wood use factor for the higher storied buildings results because the hybrid structural systems utilized need less wood.

#### Estimates of carbon benefits of mass timber use

The estimates of total carbon benefits resulting from the use of mass timber in projected four-stories-and-higher nonresidential and multifamily residential buildings were determined by separately estimating (i) the avoided emissions benefits due to the substitution of mass timber for concrete (i.e., the difference in embodied fossil GHG emissions between mass timber buildings and functionally equivalent concrete buildings), and (ii) the biogenic carbon stored in mass timber materials while in use in buildings and in landfills after the buildings are demolished after their useful service life. The estimates of the avoided emissions (S7 Table in S1 File) were based on the recently published WBLCA results [28] that provided an estimate of the embodied emissions of 8-, 12-, and 18-story functionally equivalent mass timber and reinforced concrete buildings in three U.S. regions, covering A1 (raw material extraction) to A5 (installation/construction) modules. The estimates of biogenic carbon stored in mass timber materials were based on a HWP carbon model that tracked the amount of biogenic carbon added each year as mass timber entered in new buildings, the amount of biogenic carbon discarded as buildings were demolished after their useful service life, and the biogenic carbon stored in landfills after wood materials were discarded in landfills [22, 33]. The estimates of biogenic carbon stored in these pools (in existing buildings and in landfills) were based on an exponential decay function with assumed half-life of 100 years while in use. Landfills were assumed to receive 67% of the discarded materials, and 88% of this 67% was assumed to remain indefinitely stored in landfills, while 12% of this 67% was assumed to decay with a half-life of 29 years [22, 43].

## Results

### Statistical models of nonresidential construction activities

Estimates from the final models of nonresidential floor area built are shown in S2a Table of S1 File. Most parameter estimates of these reduced-form models were statistically significantly different from zero at 5%, although the change in the real prime rate was only significant at about the 15% level. Model R^2^’s were 0.74 for the linear specification and 0.76 for the logarithmically linear specification. For each model, indicator variables for the Northeast and the South were statistically significant. Indicators for the Midwest and West were not included and so their effect was contained in the intercept. Results of model estimates indicate that most of the variation in floor area built in nonresidential structures can be explained by changes in real GDP, the prime rate of interest, and the rate of consumer price inflation. Given that result, nonresidential floor area built can be parsimoniously projected with projections of economic growth and the evolution of the costs of capital and other inputs. Models estimated for economic growth, in the form of the change in real GDP, and the real prime rate, are shown in the Supporting Information (S2b Table in S1 File). Details on how real GDP projections were aligned with SSP projections of real GDP are described in the supporting information (Appendix A in S1 File).

### Projections of new floor areas built

**Fig 1:**
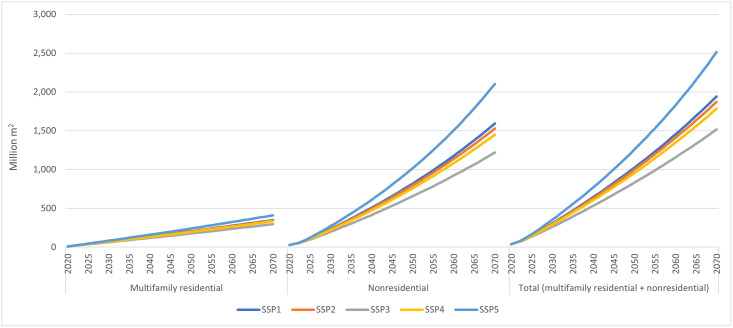
Projected new floor areas added in four-stories-and-higher multifamily residential and nonresidential buildings in the United States under different shared socioeconomic pathways (million m^2^, cumulative, 2020–2070).

The analysis suggests that the United States would likely add 7.7 to 12.8 billion m^2^ cumulatively between 2020 and 2070, representing average annual addition of 154 to 257 million m^2^ during the same period (S8 Table in S1 File). Note that the nonresidential floor areas share accounts for more than 80% of the total (S8 Table in S1 File). Accordingly, the total projected areas added cumulatively between 2020 to 2070 in four-stories-and-higher buildings categories ranged from 1.5 billion m^2^ in the lowest economic growth scenario (SSP3) to 2.5 billion m^2^ in the highest economic growth scenario (SSP5) (Fig 1 and S9 Table in S1 File), which is equivalent to average annual additions of 30.3 and 50.3 million m^2^, respectively (Table 1).

**Table 1:** Cumulative and yearly average floor areas (million m^2^) projected to be added in four-stories-and-higher multifamily residential and nonresidential buildings in the United States under median projections for each of five shared socioeconomic pathways (SSPs), 2020–2070.

Value type	Multifamily residential					Nonresidential					Total (multifamily residential + nonresidential)				
	SSP1	SSP2	SSP3	SSP4	SSP5	SSP1	SSP2	SSP3	SSP4	SSP5	SSP1	SSP2	SSP3	SSP4	SSP5
Cumulative	350	344	297	334	410	1,593	1,528	1,219	1,450	2,104	1,942	1,872	1,516	1,784	2,514
Annual	6.99	6.88	5.94	6.67	8.21	31.85	30.57	24.39	29.00	42.07	38.85	37.45	30.33	35.67	50.28

In comparison to the projected new floor areas, the average floor areas added annually in the United States between 2010 and 2018 in four-stories-and-higher buildings was about 30 million m^2^ [36, 37]. Thus, depending on the projected socioeconomic futures, our analysis depicts a small to a large increase in new floor areas added annually. The largest share of these added floor areas was projected in nonresidential buildings (more than 80%), with projected average annual new floor areas added nationally between 2020 and 2070 ranging from 24.4 to 42.1 million m^2^ (Table 1). The regional shares of multifamily, nonresidential, and total (multifamily plus nonresidential) projected new floor areas are shown in S9 Table of S1 File, which indicated that the U.S. South would add the largest total floor areas (~43%), followed by the West (~23%), the Midwest (21%) and the Northeast (~13%). These percentages were essentially the same as those projected for the four-stories-and-higher multifamily residential and nonresidential buildings (S9 Table in S1 File), consistent with their current and historical shares [36, 37].

#### Projected mass timber adoption

**Fig 2:**
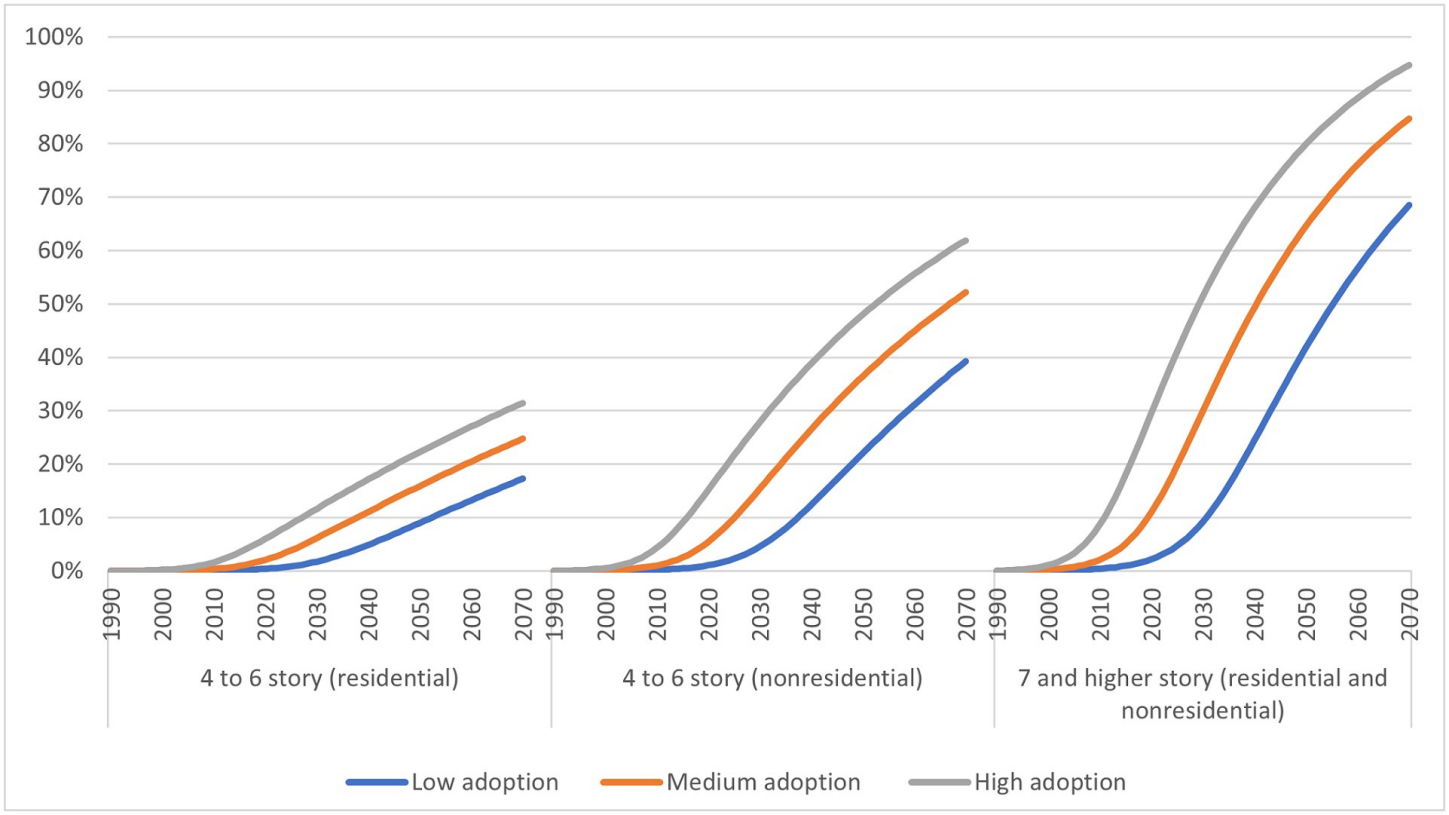
Projected mass timber market adoption levels (% relative to 100% market adoption) by building types and building heights category under low, medium, and high market adoption scenarios, 1990–2070.

We estimated the combined volumes of CLT and glulam that could go into constructing four-stories-and-higher buildings under low, medium, high, and 100% market adoption scenarios (Fig 2).

Our modeling suggested relatively slower mass timber adoption rates in four- to six-story multifamily residential buildings, with projected 17%, 25%, and 31% adoption rates by 2070 in low, medium, and high market adoption scenarios, respectively (Fig 2). In contrast, the projected mass timber adoption rates by 2070 for nonresidential buildings for the same height category were relatively faster, with low, medium, and high market adoption scenarios showing 39%, 52%, and 62% adoption rates, respectively (Fig 2). The greatest adoption rates were projected for the seven-stories-and-higher category for both nonresidential and multifamily residential buildings, suggesting that mass timber could take 69%, 84%, and 94% of its full market share by 2070, under the low, medium, and high market adoption scenarios, respectively (Fig 2).

**Fig 3:**
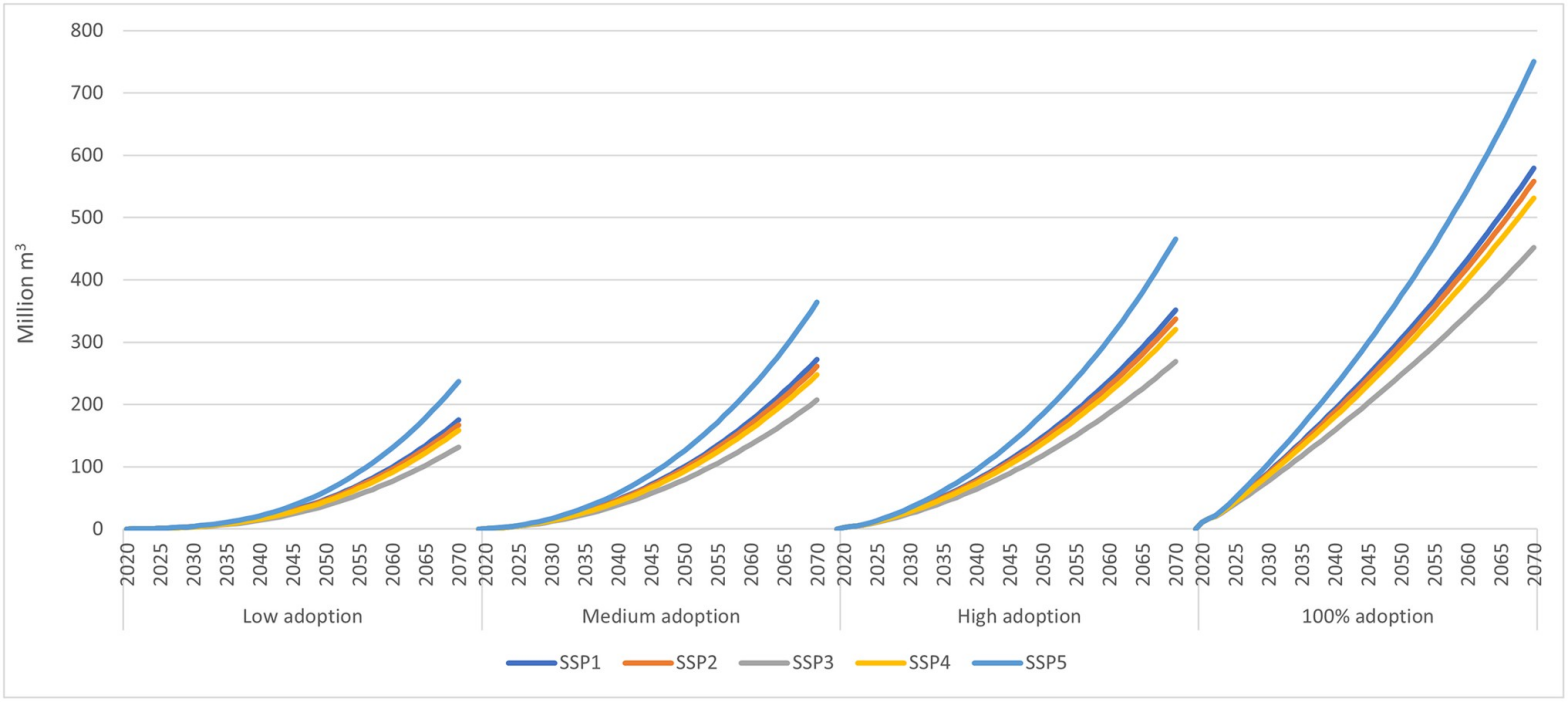
Mass timber use projected in low, medium, high, and 100% market adoption scenarios in four-stories-and-higher multifamily residential and nonresidential buildings in the United States under different shared socioeconomic pathways (million m^3^, cumulative, 2020–2070).

The U.S. aggregate potential mass timber use in four-stories-and-higher multifamily residential and nonresidential buildings in the 100% adoption scenario, from 2020 to 2070, ranged from 452 million m^3^ under SSP3 to 750 million m^3^ under SSP5 (Fig 3). This range corresponded to average annual mass timber adoption of 9.0 to 15.0 million m^3^ over the same period (Table 2).

**Table 2:** Average annual mass timber use (million m^3^) projected in low, medium, high, and 100% adoption scenarios in four-stories-and-higher residential and nonresidential buildings in the United States under different shared socioeconomic pathways (SSPs), and market adoption scenarios, 2020–2070.

Scenario	Multifamily residential					Nonresidential					Total (Multifamily residential + nonresidential)				
	SSP1	SSP2	SSP3	SSP4	SSP5	SSP1	SSP2	SSP3	SSP4	SSP5	SSP1	SSP2	SSP3	SSP4	SSP5
Low	0.39	0.38	0.33	0.37	0.46	3.11	2.96	2.31	2.80	4.28	3.50	3.34	2.63	3.17	4.74
Medium	0.63	0.62	0.53	0.60	0.74	4.82	4.60	3.61	4.35	6.54	5.45	5.22	4.14	4.95	7.28
High	0.84	0.82	0.71	0.80	0.99	6.19	5.92	4.67	5.60	8.32	7.02	6.74	5.37	6.40	9.31
100%	2.00	1.96	1.80	1.91	2.34	9.58	9.20	7.34	8.72	12.66	11.58	11.16	9.03	10.63	15.00

More than 80% of the projected increase in mass timber adoption was in nonresidential buildings (Table 2), due primarily to projected larger nonresidential floor areas in this building type (Table 1). However, as expected, the projected U.S. aggregate annual mass timber use in the low market adoption scenario was much lower and ranged from 2.6 million m^3^ in SSP3 to 4.7 million m^3^ in SSP5, representing about 29% to 32% of potential use in the 100% market adoption scenario, over the next 50 years. Note that these values represent just 1.7% to 3.1% of total U.S. lumber and wood-based panel consumption in 2022 (about 151 million m^3^) [19]. Similarly, the medium and the high adoption scenarios resulted in 4.1 to 7.3 million m^3^ and 5.4 to 9.3 million m^3^ of U.S. aggregate mass timber use annually between 2020–2070, respectively (about 45% to 48% and 59% to 63% of the 100% market adoption scenario), depending on the projected socioeconomic futures (Table 2). Regionally, the U.S. South was projected to consume the largest quantity of mass timber (S10 Table in S1 File) in all scenarios (~43%), followed by the West (~22%), Midwest (~21%) and Northeast (~13%), in line with their projected share of new floor areas added between 2020 to 2070 (S9 Table in S1 File).

#### Projected carbon benefit of mass timber adoption

**Fig 4:**
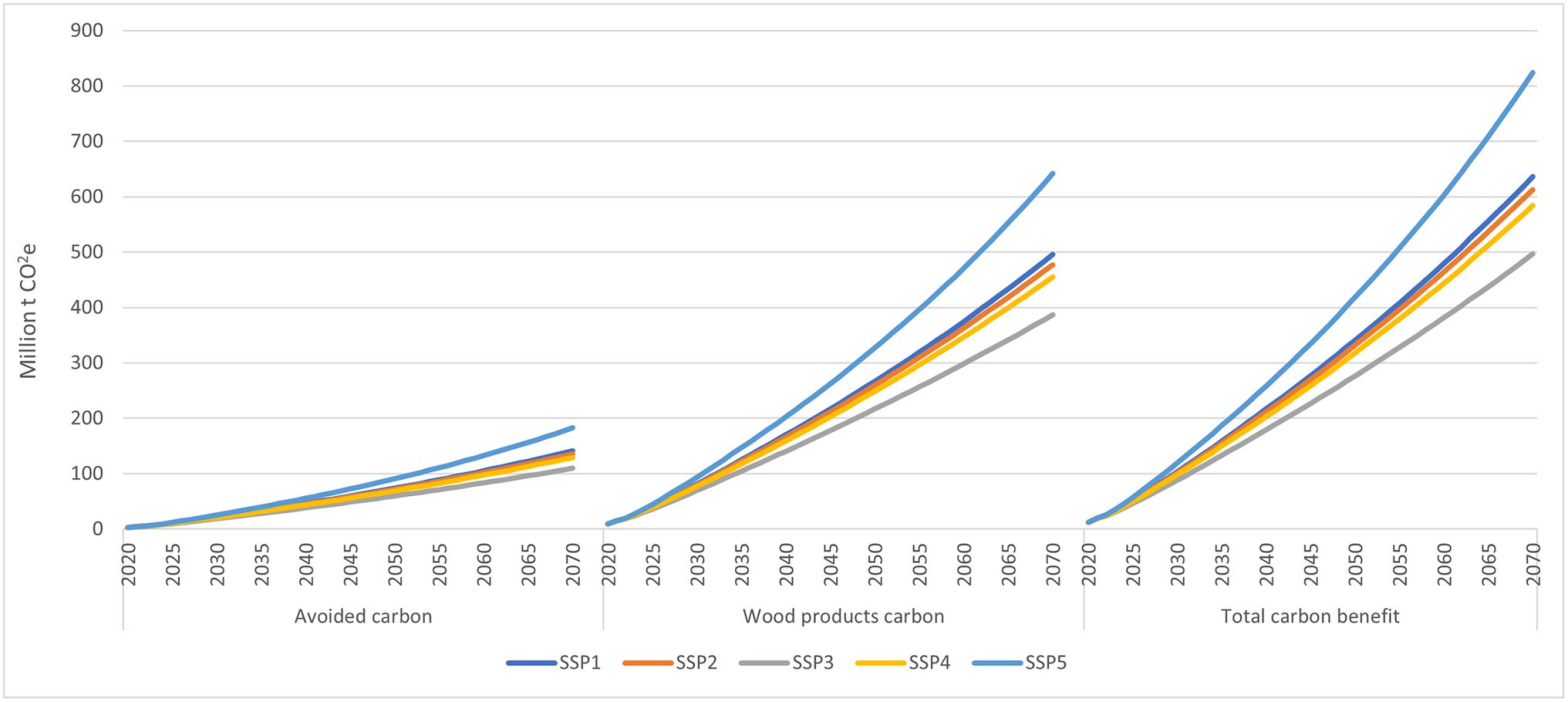
Projected carbon benefits (avoided emissions due to the substitution plus biogenic carbon stored in wood material) in the 100% market adoption scenario in four-stories-and-higher multifamily residential and nonresidential buildings in the United States under different shared socioeconomic pathways (million m^3^, cumulative, 2020–2070).

In this paper the term ‘carbon benefit’ is used to represent the LCA based global warming mitigation benefits. The estimates of total carbon reduction benefits (carbon benefit hereafter) due to use of mass timber that we report here refer to the sum of (i) avoided GHG emissions due to the substitution of mass timber for reinforced concrete structures (difference in embodied fossil GHG emissions between mass timber buildings and functionally equivalent concrete buildings), and (ii) biogenic carbon stored in mass timber materials while in use in buildings and in landfills after the buildings are demolished after their useful service. Projections of avoided emissions benefit covered raw material extraction to installation/construction modules (A1-A5). Fig 4 summarizes these estimates for a 100% market adoption scenario by SSP.

For the 100% market adoption scenario, the estimated cumulative carbon benefits of using mass timber in projected four-stories-and higher multifamily residential and nonresidential buildings from 2020 to 2070 ranged from 497 to 825 million t CO_2_e, depending on the SSP (Fig 4), giving rise to an estimated average annual carbon benefit of 9.9 to 16.5 million t CO_2_e over this period (Table 3).

**Table 3:** Estimated average annual carbon benefit (million tCO_2_e) attributable to use of mass timber (CLT and glulam) based constructions in projected four-stories-and-higher residential and nonresidential buildings in the United States under different shared socioeconomic pathways (SSPs), and market adoption scenarios, 2020–2070.

Scenario	Avoided fossil emissions					CO_2_ stored in mass timber					Total CO_2_ benefit				
	SSP1	SSP2	SSP3	SSP4	SSP5	SSP1	SSP2	SSP3	SSP4	SSP5	SSP1	SSP2	SSP3	SSP4	SSP5
Low	0.87	0.84	0.66	0.79	1.18	3.00	2.86	2.26	2.71	4.06	3.87	3.70	2.91	3.50	5.24
Medium	1.36	1.31	1.03	1.24	1.82	4.64	4.44	3.52	4.21	6.21	6.00	5.75	4.56	5.45	8.02
High	1.76	1.69	1.34	1.60	2.33	5.95	5.71	4.55	5.42	7.90	7.71	7.40	5.89	7.02	10.23
100%	2.82	2.71	2.19	2.58	3.65	9.91	9.55	7.74	9.10	12.84	12.73	12.26	9.93	11.68	16.49

These projected average annual carbon benefits represent 12% to 20% of the entire U.S. HWP carbon sequestration in 2020 (83.6 million metric tons of CO_2_e [44]). In contrast, the estimated average annual carbon benefits for the same period for the low, medium, and high market adoption scenarios ranged from 2.9 to 5.2 million t CO_2_e, 4.6 to 8.0 million t CO_2_e, and 5.9 to 10.2 million t CO_2_e, respectively depending on the SSP. Thus, depending on the scenario, use of mass timber in four-stories-and higher multifamily residential and nonresidential buildings could add 4% to 20% to current U.S. annual HWP carbon sequestration [44]. More than 78% these benefits came from biogenic carbon stored in buildings using long-lived mass timber materials, with avoided embodied emissions due to substitution for non-wood products contributing about a quarter (Table 3). Similarly, the largest share of these benefits was provided by nonresidential buildings (77% to 90%, depending on the SSP and market adoption scenarios; S11 Table in S1 File), due primarily to projected larger floor areas in those building types (S8 and S9 Tables in S1 File) coupled with relatively higher projected mass timber adoption rates (Fig 2).

## Discussion

The main finding of our analysis is that expanding mass timber usage in new mid- to high-rise buildings in the United States could provide annual carbon benefits of up to an equivalent of 20% of the current total of U.S. HWP annual carbon storage [44]. Several factors determine this result including: 1) LCA results on the carbon footprint of mass timber products, 2) building height categories and their share in total buildings, 3) projected growth in multifamily residential units vs single-family residential units, and 4) projected mass timber adoption rates.

Published LCA results indicate that manufacturing of mass timber results in extra carbon emissions compared to lumber and wood panels. Extra emissions are due to the transport of materials (e.g., lumber) to CLT and glulam facilities, the additional drying as needed to manufacture these mass timber products, and the additional energy and material inputs needed for cutting finger joints, gluing, pressing and other processes [45], leading to more fossil GHG emissions than conventional wood products [46, 47].

With respect to building heights, our analysis of potential mass timber adoption considers only four-stories-and-higher buildings, based on the premise that conventional light-framed construction would be financially more feasible in low-rise buildings. In some lower story nonresidential constructions, such as schools, warehouses, or amphitheaters, mass timber could be an attractive alternative. However, the uniqueness and variations in designs in these kinds of applications make modeling material usage at a macro level unreliable. So, we adopted a conservative approach, only considering four-stories-and-higher buildings in our assessments. In addition, our analysis assumed that the current proportion of building heights will remain unchanged in the future, which indicated that only about 20% of both nonresidential and multifamily residential buildings floor areas were occupied by four-stories-and higher buildings. However, the proportion of these types of building could increase in the future. Similarly, buildings lower than four stories could also see increased usage of mass timber in the future due to shifts in consumer preference or higher builder profitability, suggesting a theoretical possibility of increasing carbon benefits five-fold, compared to those estimated here. However, use of mass timber in all building heights may not be currently economically feasible, even if technically possible. Economic incentives such as government subsidies, tax incentives, or other market-based incentives (e.g., offset credits) for mass timber construction could encourage builders to adopt more mass timber in those building types [21].

With regard to multifamily residential units ([22], S4 Table in S1 File), projections of new floor areas added in these building types (S9 Table in S1 File) were relatively low compared to nonresidential construction. For example, the median average annual floor areas projected to be added in four-stories-and-higher multifamily residential units over the time span from 2020 to 2070 were 5.94 to 8.21 million m^2^ (S9 Table in S1 File). These values were about one-fourth to one-fifth the size of the median projected floor areas in nonresidential buildings (24.4 to 42.1 million m^2^, annually, from 2020 to 2070). In addition, the projected adoption of mass timber in four- to-six-story multifamily residential buildings was relatively lower than for the nonresidential buildings in the same height category (Fig 2).

Finally, adoption rates were projected to be lower in four-to-six-story multifamily residential and nonresidential buildings compared to taller buildings. Mass timber provides design advantages suitable for nonresidential areas, resulting in relatively higher adoption rates. Mass timber could become the most commonly used structural material for higher building height categories, due to its design and environmental benefits. The projected S-shaped pattern of the adoption diffusion curves (Fig 2) is a result of information and technology dissemination functions developed by Bass [48] and later contextualized for the wood products industry [39, 49].

Another main finding of this study is our estimated carbon benefit per unit of extra wood used in buildings (e.g., see [50–52]). Dividing the total estimated cumulative carbon benefits (avoided emissions plus biogenic carbon storage in mass timber) by the total additional mass timber volume between 2020–2070, generates a displacement factor of 1.1 t CO_2_e per m^3^ of mass timber. In other words, each additional cubic meter of mass timber that is used in new mid- to high-rise buildings in the U.S. results in carbon benefits of 1.1 t CO_2_e, on average, during 2020–2070 (or about 1.27 t CO_2_e per t CO_2_e in additional mass timber material). Such an estimated displacement factor of mass timber is a new key finding from this study and would be useful in estimating an average carbon benefit of a mass timber building constructed in the United States.

Note that our analysis of avoided emissions includes only the production and construction stages (modules A1 to A5) but not the use (B) or end-of-life (C) stages. But the estimates of biogenic carbon stored in mass timber includes carbon stored during the use stage (typically considered in module D of WBLCA) and a partial analysis of the end-of-life stage by estimating carbon remaining stored in landfills. The end-of-life stage of a building begins when the building is dismantled by demolition or deconstruction and is not intended to have any further use [53]. The displacement factor would differ (likely increase) if the end-of-life included discarded mass timber were recycled/reused or burned to substitute for fossil energy.

We also note that our estimated displacement factor of mass timber use in mid- to high-rise buildings is lower than displacement factors estimated in previous studies for conventional wood products in low- to mid-rise buildings (e.g., [50–52]). For example, the estimated average displacement factor of wood products used in structural construction was 1.3 t CO_2_e /t CO_2_e, based on the review by Leskinen et al. [50] of 51 studies worldwide. Similarly, the average estimated displacement factor based on the review by Sathre and O’Connor [52] of 21 studies worldwide was 2.1 t CO_2_e /t CO_2_e. For the United States, Nepal et al. [51] estimated an average U.S. aggregate displacement factor of 2.83 t CO_2_e /t CO_2_e for softwood lumber projected to be used in low- to mid-rise nonresidential construction over the 50-year period. Our relatively lower displacement factor of mass timber (1.1 t CO_2_e /t CO_2_e), compared to conventional wood products use in low- to mid-rise buildings, is due to (i) lower estimated avoided emissions benefits of mass timber, because of the additional processing and transport required, and (ii) lower mass timber use intensity (volume of mass timber per square meter) compared to conventional low rise structural light-framed buildings based on building code requirements. For example, the average wood usage intensity for mass timber assumed in this study, based on Dolan et al. [42], 0.27 m^3^/m^2^, was about one-third less than the average wood use intensity in light-framed residential buildings in the United States (~ 0.41 m^3^/m^2^, [54, 55]). However, both the estimates of displacement factors in the past literature for traditional wood products, and in this study for mass timber, are associated with large uncertainties stemming from differing assumptions, methodological approaches, and inherent uncertainties associated with modeling the future. We discuss below key factors that could affect our analysis and add further uncertainty to our results.

The estimated total carbon benefits of mass timber and the displacement factor reported in this study are based on an assumption that the extra use of wood for mass timber from a sustainably managed forest would recover any reduction in forest biomass (and carbon) due to increased harvests through biological growth over time. In other words, over a sufficiently long time horizon, there is no net emission of carbon from forests due to wood harvests [56, 57]. However, the carbon lost due to additional harvesting can take decades to be recovered through forest regrowth [58, 59]. Also, the true carbon benefits (and displacement factor) of increased mass timber use in new buildings will depend on where timber is harvested, how the biophysical growth of forest changes in harvest regions, where mass timber is manufactured, where wood materials needed for its manufacture are sourced, and how forest product markets and the forest sector respond to changes in demand for mass timber. Forests and the wood products sector would respond by changing products prices and production, consumption, and trade of both traditional wood products (e.g., sawlogs, lumber) and mass timber products. All these potential dynamics in the forest sector would add uncertainty to our estimated results, especially to the estimates of the displacement factor. Subsequent effects of increased mass timber adoption, including changes in forest rent, land use, forest management, forest growth, and standing inventory, could be revealed by integrating a global market equilibrium model of the forest sector with a detailed forest ecosystem model and a land use model.

Along these lines, we sought to obtain preliminary insights into the likely long-term effects of mass timber adoption on U.S. aggregate forest harvests, inventory, and growth-to-drain ratios (an indicator of forest sustainability). To do this, we incorporated our 100% market adoption scenario for mass timber into a side analysis and projected its impacts on the forest sector through 2070. Among the outputs of this exercise were projected impacts of high mass timber adoption on domestic U.S. harvests and standing stocks of timber (inventory). Given an assumption that the current U.S. aggregate average net annual forest growth rate would remain same (708.18 million m^3^ in 2017, [60]), we found that our projected mass timber adoption would lead to an average increase in U.S. annual timber removals of 7% to 13% over the years 2020 to 2070, compared to 2017 removals (369 million m^3^, [60]), depending on the SSP (S12 Table in S1 File). Such a projected increase in removals would lead to a reduction in forest inventory by 2.0% to 3.2% by 2070. In other words, more than 97% of the projected reduction in timber inventory due to increased harvest for mass timber would be offset by 2070 by biological forest growth (relative to the case where no new demand for wood occurred), even if the average U.S. aggregate timber growth rate remained at the 2017 level. However, there are several reasons why this forest growth rate could increase in the future and could offset projected reductions in timber inventory by 2070. For example, increased harvests would lead to reduced forest density (e.g., younger forests), which would tend to raise stand growth rates [61]. Similarly, increased demand for wood products (mass timber) would lead to increased timber prices, ceteris paribus [17, 62], which could provide a market incentive to invest in forest management activities such as thinning or the establishment of new fast-growing pine plantations. Finally, this preliminary analysis found that the projected increase in harvests in a 100% adoption scenario would lead to slight declines in average growth-to-drain ratios during 2020–2070, ranging from 5% to 8%, depending on SSPs, compared to a 2017 growth-to-drain ratio of 2.14. These outcomes suggest that the current U.S. annual forest growth rate is high enough to sustain the additional harvests needed to meet the projected production of mass timber domestically (a growth-to-drain ratio of 1 means that annual harvest is equal to annual removal). However, the precise impact on these forest variables will depend on complex biophysical and market interactions that the research team is planning to investigate in the near future.

There are some additional factors that could add further uncertainty to our results. Our study did not consider the operational energy usage, which is accounted in building use stage of the WBLCA (module B). While several studies assume the same operational energy between mass timber and functionally equivalent alternative buildings [25, 63, 64], others suggest that energy consumption is lower in mass timber buildings, due to their better thermal properties [65], an important research topic that needs further investigation. Our analysis provides a partial analysis of end-of-life options by estimating biogenic carbon storage in landfills after demolition of mass timber buildings, which occurs outside the building system boundary or beyond-life (module D) [53]. Alternate end-of-life options other than landfilling exist, such as recycling, burning with energy capture, and composting [66]. These options, such as increased recycling/reuse of demolished or deconstructed wood materials from mass timber construction (e.g., see [22]) or burning discarded wood to substitute for fossil fuels, may increase the overall carbon benefits of mass timber. However, the degrees and the magnitudes of carbon benefits resulting from alternative end-of-life options of mass timber material are yet to be quantified unlike the case for more conventional wood material (e.g., [67]).

## Conclusions

Results from this study lead to several conclusions regarding projected demand for nonresidential and residential floor areas and anticipated carbon benefits of using mass timber in construction. First, about 154 and 257 million m^2^ of new floor areas would be added annually in the United States over the fifty years spanning 2020–2070, depending on the SSP. More than 80% of those added floor areas would be shared by nonresidential buildings, and about 20% of all new multifamily residential and nonresidential floor areas would be shared by higher than three-story buildings (about 30 to 50 million m^2^), based on the current proportion of those building heights. Second, the adoption of mass timber usage in new buildings would vary between 2.6 and 4.7 million m^3^/yr (under the low adoption scenario) and 9.0 to 15.0 million m^3^/yr (the 100% adoption scenario), depending on the SSP, on average, during 2020–2070. Third, the use of mass timber instead of concrete in new buildings that are higher than three stories would lead to projected carbon benefits of 2.9 to 5.2 million t CO_2_e/yr (the low adoption scenario) to between 9.9 and 16.5 million t CO_2_e/yr (the 100% adoption scenario), which included avoided emissions benefits due to substitution of non-wood materials and biogenic carbon stored in mass timber materials, during 2020–2070, with avoided emissions comprising 22% and carbon stored in mass timber materials comprising 78% of total projected carbon benefits. And fourth, the average displacement factor of mass timber, considering cumulative avoided emissions and wood carbon storage benefits during 2020–2070, was 1.1 t CO_2_e per m^3^, which we can describe as the marginal carbon benefit of 1.1 t CO_2_e for every extra m^3^ of mass timber used in new mid-to high-rise mass timber construction in the United States.

Our analysis indicates that, with favorable public policies and removal of barriers to market adoption in the future, it would be possible to increase carbon benefits five-fold, compared to current levels reported in this study, which is based on mass timber use in four-stories-and-higher buildings. However, the true, likely higher, carbon benefits of mass timber use will also depend on how future demand for mass timber affects domestic and international wood products markets (e.g., prices, harvests, production, consumption, and trade of wood products), where mass timber is manufactured, and how forest land use and forest management respond to changes in markets. The benefits will also depend on additional possible end-of-life options after demolition of mass timber buildings. Such factors can be included by investigating biophysical and market interactions in the forest sector, supplemented by a complete WBLCA covering all stages, which our research team aims to investigate in the near future.

## Supporting information

S1 File (DOCX)

S1 Data (XLSX)
