# Supplementary material for: The potential use of mass timber in mid-to high-rise construction and the associated carbon benefits in the United States
Source: PLoS One. 2024 Mar 20;19(3):e0298379. doi: 10.1371/journal.pone.0298379 (PMC10954139; doi:10.1371/journal.pone.0298379)
Supplement: S1 File — (DOCX) [file pone.0298379.s001.docx]

Supporting tables, figures, appendices, and references

**Table S1.** Data sources and descriptive statistics of data used in estimating econometric models (expressed as multiyear averages, which were inputs to the econometric estimation).

| Variables | Units | Obser- vations | Mean | Standard deviation | Min | Max | Data Source |
| --- | --- | --- | --- | --- | --- | --- | --- |
| Floor Area Built, Northeast | Million ft^2^ | 11 | 166.40 | 42.33 | 95.33 | 215.75 | [1] |
| Floor Area Built, Midwest | Million ft^2^ | 11 | 279.17 | 90.47 | 184.00 | 468.00 | [1] |
| Floor Area Built, South | Million ft^2^ | 11 | 527.92 | 166.23 | 250.00 | 710.00 | [1] |
| Floor Area Built, West | Million ft^2^ | 11 | 284.45 | 90.48 | 136.67 | 487.00 | [1] |
| Real GDP, Northeast | Million US $ | 12 | 2,500,251 | 713,572 | 1,437,810 | 3,528,193 | [2] |
| Real GDP, Midwest | Million US $ | 12 | 2,540,721 | 693,766 | 1,605,223 | 3,518,737 | [2] |
| Real GDP, South | Million US $ | 12 | 3,851,151 | 1,434,165 | 1,894,348 | 6,030,432 | [2] |
| Real GDP, West | Million US $ | 12 | 2,662,745 | 1,018,039 | 1,256,716 | 4,309,445 | [2] |
| Prime Rate | Percent | 12 | 3.94 | 2.17 | 0.68 | 7.04 | [3] |
| Consumer Price Index | Index | 12 | 156.99 | 56.96 | 59.75 | 240.49 | [4] |
| Construction Earnings | $/week | 12 | 631.15 | 229.35 | 275.55 | 1,021.23 | [5] |
| Residential Construction Earnings | $/week | 8 | 612.30 | 143.39 | 426.96 | 831.73 | [6] |
| Nonresidential Construction Earnings | $/week | 8 | 803.63 | 212.91 | 534.99 | 1,110.27 | [7] |
| Concrete PPI | Index | 12 | 126.83 | 94.47 | 38.07 | 321.17 | [8] |
| Net Immigration^1^ | Per 1,000  Population | 12 | 976,888 | 289,756 | 673,517 | 1,622,406 | [9] |

^1^ Used as an instrument in instrumental variables equations estimates.

**Table S2a.** Estimated parameters of econometric model of nonresidential buildings floor area built (in millions of square feet units) in the United States, based on periodic average annual data, 1983-2020.

| Model 1: Floor Area Built | |  |  | Model 2: ln(Floor Area Built) | |  |  |
| --- | --- | --- | --- | --- | --- | --- | --- |
|  | Coefficient | Std. Err | t-value |  | Coefficient | Std. Err | t-value |
| Northeast | -103.68 | 20.24 | -5.12 | Northeast | -0.48 | 0.08 | -5.72 |
| South | 179.99 | 53.40 | 3.37 | South | 0.56 | 0.12 | 4.79 |
| Change in regional real GDPt-1 | 0.000703 | 0.000331 | 2.12 | Change in ln(real regional GDPt-1) | 6.45 | 3.42 | 1.89 |
| Change in regional real GDPt-2 | 0.000931 | 0.000399 | 2.33 | Change in ln(real regional GDPt-2) | 9.45 | 4.03 | 2.34 |
| Change in real prime ratet | -27.20 | 19.32 | -1.41 | Change in ln(CPIt) | 8.32 | 4.16 | 2.00 |
| Change in real prime ratet-1 | 46.17 | 28.44 | 1.62 | ln(yeart) | 33.27 | 15.91 | 2.09 |
| Constant | 176.24 | 31.80 | 5.54 | Constant | -247.91 | 121.14 | -2.05 |
| Observations | 44 |  |  | Observations | 44 |  |  |
| R2 | 0.74 |  |  | R2 | 0.76 |  |  |
| Root Mean Squared Error | 92.87 |  |  | Root Mean Squared Error | 0.27 |  |  |

**Table S2b.** Parameters estimated from models of regional real gross domestic product (GDP) and the real prime rate of interest, based on annual data, 1983-2020.

| Dependent Variable | Regressors | Coefficient | Std. Err. | t-value | R^2^ | Observations |
| --- | --- | --- | --- | --- | --- | --- |
| Change in Northeast real GDP_t_ | Change in Northeast real GDP_t-1_ | 0.47 | 0.17 | 2.85 | 0.17 | 38 |
|  | Constant | 0.011 | 0.0048 | 2.25 |  |  |
|  |  |  |  |  |  |  |
| Change in Midwest real GDP_t_ | Change in Midwest real GDP_t-1_ | 0.23 | 0.16 | 1.45 | 0.05 | 38 |
|  | Constant | 0.016 | 0.0058 | 2.73 |  |  |
|  |  |  |  |  |  |  |
| Change in South real GDP_t_ | Change in South real GDP_t-1_ | 0.49 | 0.16 | 3.08 | 0.20 | 38 |
|  | Constant | 0.014 | 0.0060 | 2.32 |  |  |
|  |  |  |  |  |  |  |
| Change in West real GDP_t_ | Change in West real GDP_t-1_ | 0.36 | 0.14 | 2.56 | 0.11 | 38 |
|  | Constant | 0.019 | 0.0056 | 3.48 |  |  |
|  |  |  |  |  |  |  |
| Real Prime Rate_t_ | Real Prime Rate_t-1_ | 0.76 | 0.071 | 10.69 | 0.66 | 38 |
|  | Constant | 0.83 | 0.36 | 2.32 |  |  |

**Table S3. Historical and projected^1^ average floor space (square meters) per unit of multifamily residential buildings by U.S. regions.** Data source: US Census Bureau [10].

|  | Historical | | | | | Projected based on historical linear trends | | | | |
| --- | --- | --- | --- | --- | --- | --- | --- | --- | --- | --- |
| Region | 1980 | 1990 | 2000 | 2010 | 2020 | 2030 | 2040 | 2050 | 2060 | 2070 |
| Northeast | 91 | 98 | 107 | 121 | 98 | 120 | 125 | 130 | 135 | 139 |
| Midwest | 87 | 98 | 101 | 106 | 102 | 124 | 131 | 139 | 146 | 153 |
| South | 93 | 90 | 106 | 110 | 109 | 122 | 129 | 135 | 141 | 148 |
| West | 90 | 93 | 100 | 103 | 98 | 112 | 117 | 121 | 126 | 130 |
| U.S. | 90 | 95 | 103 | 110 | 102 | 120 | 125 | 131 | 137 | 143 |

^1^ Based on linear trends of historical average floor space data, 1974-2020 [10].

**Table S4. Median number of multifamily residential starts (thousand units) for U.S. aggregate region under alternative shared socioeconomic pathways (SSP).** (Source: Prestemon et al. [11].

| SSPs | 2020 | 2025 | 2030 | 2035 | 2040 | 2045 | 2050 | 2055 | 2060 | 2065 | 2070 |
| --- | --- | --- | --- | --- | --- | --- | --- | --- | --- | --- | --- |
| SSP1 | 340 | 268 | 266 | 260 | 257 | 255 | 255 | 259 | 256 | 248 | 252 |
| SSP2 | 334 | 264 | 257 | 253 | 256 | 256 | 251 | 255 | 256 | 253 | 249 |
| SSP3 | 322 | 230 | 225 | 220 | 217 | 216 | 217 | 219 | 215 | 211 | 212 |
| SSP4 | 331 | 256 | 250 | 245 | 249 | 248 | 243 | 247 | 248 | 243 | 241 |
| SSP5 | 357 | 309 | 314 | 310 | 304 | 301 | 306 | 312 | 305 | 298 | 301 |

**Table S5. Share of residential structure and floor area by building story.** (Source: [12], multifamily residential buildings, and [1], nonresidential buildings).

| Building story | | | | | | | |
| --- | --- | --- | --- | --- | --- | --- | --- |
|  | 1 | 2 | 3 | 4 to 9 | 10 or more | 4 to 6* | 6 or more* |
| Nonresidential floor area | 45% | 24% | 11% | 13% | 6% | 7.5% | 11.7% |
| Building story | | | | | | | |
|  | 1 | 2 | 3 | 4 to 6 | 6 or more |  |  |
| Residential structure^ | 12% | 40% | 27% | 13% | 8% |  |  |

* Author calculated numbers using nonlinear interpolation technique

^ Averages of 2015, 2017, and 2019

**Table S6. Intensity of mass timber use (m^3^/m^2^) applied in projecting mass timber demand in this study.** (Source: Dolan et al. [13]).

| Mass timber building height | CLT | Glulam | CLT+ Glulam |
| --- | --- | --- | --- |
| 4 to 6 stories | 0.21 | 0.05 | 0.26 |
| 7 to 12 stories | 0.27 | 0.06 | 0.33 |
| 13 and higher stories | 0.17 | 0.07 | 0.24 |

**Table S7. Estimated average embodied emissions of concrete and mass timber buildings (kg CO2e/m^2^), average floor space (m^2^), and average mass timber volume (m^3^) used in calculating A1-A5 stage avoided carbon emissions benefit per m^3^ of mass timber used in multifamily residential and nonresidential buildings.**

| US Regions^*^ | | | | | |
| --- | --- | --- | --- | --- | --- |
|  | PNW | NE | SE | MW | US |
| Building type | A1-A5 embodied emissions (kg CO2e/m^2^) | | | |  |
| Mass timber | 151.23 | 132.13 | 150.77 | 144.71 | 144.71 |
| Concrete | 248.77 | 229.40 | 225.73 | 234.63 | 234.63 |
| Avoided embodied emissions due to mass timber use (kg CO2e/m^2^) | | | | | |
|  | 97.53 | 97.27 | 74.97 | 89.92 | 89.92 |
| Total floor space (m^2^) | | | | | |
| Mass timber | 15,004 | 15,004 | 15,004 | 15,004 | 15,004 |
| Concrete | 15,004 | 15,004 | 15,004 | 15,004 | 15,004 |
| Total mass timber volume (m^3^) | | | | | |
| Mass timber | 4,778 | 5,524 | 5,522 | 5,275 | 5,275 |
| Concrete | 0 | 0 | 0 | 0 | 0 |
|  | Avoided embodied emissions due to mass timber use (kg CO2e/m^3^) | | | | |
|  | -306.27 | -264.18 | -203.68 | -255.78 | -255.78 |

Notes: Values represent the average of 8, 12, and 18 story buildings reported in Puettmann et al. [14].

^*^ PNW = Pacific Northwest; NE = Northeast, SE = Southeast, MW = Midwest. The values for MW and US are the averages of values for the PNW, NE, and SE reported in Puettmann et al. [14].

**Table S8. Cumulative and yearly average floor space area (million m^2^) projected to be added in all building height categories of multifamily residential and nonresidential buildings in the United States by U.S. regions under five shared socioeconomic pathways, 2020-2070.**

|  | Multifamily residential | | | | | Nonresidential | | | | | Multifamily residential + nonresidential | | | | | | |
| --- | --- | --- | --- | --- | --- | --- | --- | --- | --- | --- | --- | --- | --- | --- | --- | --- | --- |
|  | Cumulative value, 2020-2070 (million m^2^) | | | | | | | | | | | | | |  | |  | |
| Region | SSP1 | SSP2 | SSP3 | SSP4 | SSP5 | SSP1 | SSP2 | SSP3 | SSP4 | SSP5 | SSP1 | SSP2 | SSP3 | SSP4 | | SSP5 | |
| Northeast | 194 | 193 | 151 | 184 | 245 | 1,082 | 1,028 | 789 | 967 | 1,466 | 1,276 | 1,221 | 940 | 1,152 | | 1,711 | |
| Midwest | 312 | 299 | 265 | 290 | 365 | 1,778 | 1,712 | 1,387 | 1,633 | 2,305 | 2,090 | 2,011 | 1,651 | 1,923 | | 2,670 | |
| South | 723 | 707 | 630 | 689 | 828 | 3,566 | 3,429 | 2,754 | 3,252 | 4,703 | 4,289 | 4,136 | 3,384 | 3,942 | | 5,530 | |
| West | 380 | 383 | 321 | 371 | 449 | 1,868 | 1,791 | 1,420 | 1,698 | 2,481 | 2,248 | 2,174 | 1,741 | 2,069 | | 2,931 | |
| US Total** | 1,609 | 1,581 | 1,367 | 1,535 | 1,887 | 8,294 | 7,960 | 6,350 | 7,551 | 10,956 | 9,903 | 9,542 | 7,717 | 9,086 | | 12,843 | |
| Average annual value, 2020-2070 (million m^2^) | | | | | | | | | | | | | | | | | | |
| Region | SSP1 | SSP2 | SSP3 | SSP4 | SSP5 | SSP1 | SSP2 | SSP3 | SSP4 | SSP5 | SSP1 | SSP2 | SSP3 | SSP4 | | SSP5 | |
| Northeast | 3.87 | 3.86 | 3.03 | 3.69 | 4.90 | 21.64 | 20.57 | 15.78 | 19.35 | 29.33 | 25.52 | 24.43 | 18.81 | 23.04 | | 34.23 | |
| Midwest | 6.24 | 5.97 | 5.29 | 5.80 | 7.30 | 35.56 | 34.24 | 27.74 | 32.67 | 46.10 | 41.80 | 40.21 | 33.03 | 38.47 | | 53.40 | |
| South | 14.46 | 14.14 | 12.60 | 13.79 | 16.55 | 71.32 | 68.57 | 55.08 | 65.05 | 94.06 | 85.78 | 82.71 | 67.68 | 78.83 | | 110.61 | |
| West | 7.60 | 7.66 | 6.41 | 7.42 | 8.99 | 37.36 | 35.83 | 28.40 | 33.96 | 49.63 | 44.96 | 43.49 | 34.82 | 41.38 | | 58.62 | |
| US Total** | 32.17 | 31.63 | 27.33 | 30.69 | 37.74 | 165.88 | 159.21 | 127.01 | 151.02 | 219.11 | 198.05 | 190.84 | 154.34 | 181.72 | | 256.85 | |
| Regional share (%) | | | | | | | | | | | | | | | | | | |
| Region | SSP1 | SSP2 | SSP3 | SSP4 | SSP5 | SSP1 | SSP2 | SSP3 | SSP4 | SSP5 | SSP1 | SSP2 | SSP3 | SSP4 | | SSP5 | |
| Northeast | 12 | 12 | 11 | 12 | 13 | 13 | 13 | 12 | 13 | 13 | 13 | 13 | 12 | 13 | | 13 | |
| Midwest | 19 | 19 | 19 | 19 | 19 | 21 | 22 | 22 | 22 | 21 | 21 | 21 | 21 | 21 | | 21 | |
| South | 45 | 45 | 46 | 45 | 44 | 43 | 43 | 43 | 43 | 43 | 43 | 43 | 43 | 43 | | 43 | |
| West | 24 | 24 | 23 | 24 | 24 | 23 | 23 | 22 | 22 | 23 | 22 | 23 | 22 | 23 | | 23 | |

* Based on the projected multifamily residential units in [11] and linear trends of historical average floor space data, 1974-2020 [10].

** The sum of the regional values does not exactly equal values shown for the U.S. total for multifamily residential construction because the U.S. total values were obtained from a separate national level econometric model for multifamily residential units.

**Table S9. Cumulative and yearly average floor areas (million m^2^) projected to be added in four-stories-and-higher multifamily residential and nonresidential building categories by U.S. regions under different shared socioeconomic pathways (SSPs), 2020-2070.**

|  | Multifamily residential | | | | | Nonresidential | | | | | Multifamily residential + nonresidential | | | | | | |
| --- | --- | --- | --- | --- | --- | --- | --- | --- | --- | --- | --- | --- | --- | --- | --- | --- | --- |
|  | Cumulative value, 2020-2070 (million m^2^) | | | | | | | | | | | | | |  | |  | |
| Region | SSP1 | SSP2 | SSP3 | SSP4 | SSP5 | SSP1 | SSP2 | SSP3 | SSP4 | SSP5 | SSP1 | SSP2 | SSP3 | SSP4 | | SSP5 | |
| Northeast | 42 | 42 | 33 | 40 | 53 | 208 | 197 | 152 | 186 | 282 | 250 | 239 | 184 | 226 | | 335 | |
| Midwest | 68 | 65 | 58 | 63 | 79 | 341 | 329 | 266 | 314 | 443 | 409 | 394 | 324 | 377 | | 522 | |
| South | 157 | 154 | 137 | 150 | 180 | 685 | 658 | 529 | 624 | 903 | 842 | 812 | 666 | 774 | | 1,083 | |
| West | 83 | 83 | 70 | 81 | 98 | 359 | 344 | 273 | 326 | 476 | 441 | 427 | 342 | 407 | | 574 | |
| US Total** | 350 | 344 | 297 | 334 | 410 | 1,593 | 1,528 | 1,219 | 1,450 | 2,104 | 1,942 | 1,872 | 1,516 | 1,784 | | 2,514 | |
| Average annual value, 2020-2070 (million m^2^) | | | | | | | | | | | | | | | | | | |
| Region | SSP1 | SSP2 | SSP3 | SSP4 | SSP5 | SSP1 | SSP2 | SSP3 | SSP4 | SSP5 | SSP1 | SSP2 | SSP3 | SSP4 | | SSP5 | |
| Northeast | 0.84 | 0.84 | 0.66 | 0.80 | 1.07 | 4.16 | 3.95 | 3.03 | 3.72 | 5.63 | 5.00 | 4.79 | 3.69 | 4.52 | | 6.70 | |
| Midwest | 1.36 | 1.30 | 1.15 | 1.26 | 1.59 | 6.83 | 6.57 | 5.33 | 6.27 | 8.85 | 8.18 | 7.87 | 6.48 | 7.53 | | 10.44 | |
| South | 3.14 | 3.07 | 2.74 | 3.00 | 3.60 | 13.69 | 13.17 | 10.58 | 12.49 | 18.06 | 16.84 | 16.24 | 13.32 | 15.49 | | 21.66 | |
| West | 1.65 | 1.67 | 1.39 | 1.61 | 1.95 | 7.17 | 6.88 | 5.45 | 6.52 | 9.53 | 8.83 | 8.54 | 6.85 | 8.13 | | 11.48 | |
| US Total** | 6.99 | 6.88 | 5.94 | 6.67 | 8.21 | 31.85 | 30.57 | 24.39 | 29.00 | 42.07 | 38.85 | 37.45 | 30.33 | 35.67 | | 50.28 | |
| Regional share (%) | | | | | | | | | | | | | | | | | | |
| Region | SSP1 | SSP2 | SSP3 | SSP4 | SSP5 | SSP1 | SSP2 | SSP3 | SSP4 | SSP5 | SSP1 | SSP2 | SSP3 | SSP4 | | SSP5 | |
| Northeast | 12 | 12 | 11 | 12 | 13 | 13 | 13 | 12 | 13 | 13 | 13 | 13 | 12 | 13 | | 13 | |
| Midwest | 19 | 19 | 19 | 19 | 19 | 21 | 22 | 22 | 22 | 21 | 21 | 21 | 21 | 21 | | 21 | |
| South | 45 | 45 | 46 | 45 | 44 | 43 | 43 | 43 | 43 | 43 | 43 | 43 | 43 | 43 | | 43 | |
| West | 24 | 24 | 23 | 24 | 24 | 23 | 23 | 22 | 22 | 23 | 22 | 23 | 22 | 23 | | 23 | |

**Table S10.** **Average annual mass timber use (million m^3^) projected in low, medium, high, and 100% adoption scenarios in four-stories-and-higher multifamily residential and nonresidential buildings by U.S. regions under different shared socioeconomic pathways (SSPs) and market adoption scenarios, 2020-2070.**

|  | Multifamily residential | | | | | Nonresidential | | | | | Multifamily residential + nonresidential | | | | |
| --- | --- | --- | --- | --- | --- | --- | --- | --- | --- | --- | --- | --- | --- | --- | --- |
|  | Low mass timber adoption scenario, average annual value, 2020-2070, million m^3^ | | | | | | | | | | | | | | | |
| Region | SSP1 | SSP2 | SSP3 | SSP4 | SSP5 | SSP1 | SSP2 | SSP3 | SSP4 | SSP5 | SSP1 | SSP2 | SSP3 | SSP4 | SSP5 |
| Northeast | 0.05 | 0.05 | 0.03 | 0.04 | 0.06 | 0.42 | 0.39 | 0.29 | 0.36 | 0.58 | 0.46 | 0.43 | 0.33 | 0.41 | 0.64 |
| Midwest | 0.08 | 0.07 | 0.06 | 0.07 | 0.09 | 0.66 | 0.63 | 0.50 | 0.60 | 0.89 | 0.74 | 0.71 | 0.57 | 0.67 | 0.98 |
| South | 0.17 | 0.17 | 0.15 | 0.17 | 0.20 | 1.34 | 1.28 | 1.00 | 1.21 | 1.86 | 1.52 | 1.45 | 1.15 | 1.38 | 2.05 |
| West | 0.09 | 0.09 | 0.08 | 0.09 | 0.11 | 0.69 | 0.66 | 0.51 | 0.62 | 0.95 | 0.78 | 0.75 | 0.58 | 0.71 | 1.06 |
| US Total** | 0.39 | 0.38 | 0.33 | 0.37 | 0.46 | 3.11 | 2.96 | 2.31 | 2.80 | 4.28 | 3.50 | 3.34 | 2.63 | 3.17 | 4.74 |
| Medium mass timber adoption scenario, average annual value, 2020-2070, million m^3^ | | | | | | | | | | | | | | | | |
| Region | SSP1 | SSP2 | SSP3 | SSP4 | SSP5 | SSP1 | SSP2 | SSP3 | SSP4 | SSP5 | SSP1 | SSP2 | SSP3 | SSP4 | SSP5 |
| Northeast | 0.07 | 0.07 | 0.06 | 0.07 | 0.10 | 0.64 | 0.60 | 0.46 | 0.56 | 0.88 | 0.71 | 0.68 | 0.51 | 0.63 | 0.98 |
| Midwest | 0.12 | 0.12 | 0.10 | 0.11 | 0.15 | 1.03 | 0.98 | 0.79 | 0.94 | 1.37 | 1.15 | 1.10 | 0.89 | 1.05 | 1.51 |
| South | 0.28 | 0.28 | 0.24 | 0.27 | 0.32 | 2.08 | 1.99 | 1.57 | 1.88 | 2.83 | 2.36 | 2.27 | 1.81 | 2.15 | 3.15 |
| West | 0.15 | 0.15 | 0.12 | 0.14 | 0.17 | 1.07 | 1.02 | 0.80 | 0.97 | 1.47 | 1.22 | 1.17 | 0.92 | 1.11 | 1.64 |
| US Total** | 0.63 | 0.62 | 0.53 | 0.60 | 0.74 | 4.82 | 4.60 | 3.61 | 4.35 | 6.54 | 5.45 | 5.22 | 4.14 | 4.95 | 7.28 |
| High mass timber adoption scenario, average annual value, 2020-2070, million m^3^ | | | | | | | | | | | | | | | | |
| Region | SSP1 | SSP2 | SSP3 | SSP4 | SSP5 | SSP1 | SSP2 | SSP3 | SSP4 | SSP5 | SSP1 | SSP2 | SSP3 | SSP4 | SSP5 |
| Northeast | 0.10 | 0.10 | 0.08 | 0.09 | 0.13 | 0.82 | 0.77 | 0.59 | 0.72 | 1.12 | 0.92 | 0.87 | 0.66 | 0.82 | 1.25 |
| Midwest | 0.16 | 0.16 | 0.14 | 0.15 | 0.19 | 1.32 | 1.27 | 1.02 | 1.21 | 1.74 | 1.49 | 1.43 | 1.16 | 1.36 | 1.94 |
| South | 0.38 | 0.37 | 0.33 | 0.36 | 0.43 | 2.67 | 2.56 | 2.03 | 2.42 | 3.59 | 3.04 | 2.92 | 2.35 | 2.78 | 4.02 |
| West | 0.20 | 0.20 | 0.17 | 0.19 | 0.23 | 1.38 | 1.32 | 1.03 | 1.25 | 1.87 | 1.58 | 1.52 | 1.20 | 1.44 | 2.10 |
| US Total** | 0.84 | 0.82 | 0.71 | 0.80 | 0.99 | 6.19 | 5.92 | 4.67 | 5.60 | 8.32 | 7.02 | 6.74 | 5.37 | 6.40 | 9.31 |
| 100% mass timber adoption scenario, average annual value, 2020-2070, million m^3^ | | | | | | | | | | | | | | | | |
| Region | SSP1 | SSP2 | SSP3 | SSP4 | SSP5 | SSP1 | SSP2 | SSP3 | SSP4 | SSP5 | SSP1 | SSP2 | SSP3 | SSP4 | SSP5 |
| Northeast | 0.24 | 0.24 | 0.19 | 0.23 | 0.30 | 1.25 | 1.19 | 0.91 | 1.12 | 1.69 | 1.49 | 1.43 | 1.10 | 1.35 | 2.00 |
| Midwest | 0.39 | 0.37 | 0.33 | 0.36 | 0.45 | 2.05 | 1.98 | 1.60 | 1.89 | 2.66 | 2.44 | 2.35 | 1.93 | 2.25 | 3.12 |
| South | 0.90 | 0.88 | 0.78 | 0.86 | 1.03 | 4.12 | 3.96 | 3.18 | 3.76 | 5.43 | 5.02 | 4.84 | 3.96 | 4.61 | 6.46 |
| West | 0.47 | 0.48 | 0.40 | 0.46 | 0.56 | 2.16 | 2.07 | 1.64 | 1.96 | 2.87 | 2.63 | 2.55 | 2.04 | 2.42 | 3.43 |
| US Total** | 2.00 | 1.96 | 1.80 | 1.91 | 2.34 | 9.58 | 9.20 | 7.34 | 8.72 | 12.66 | 11.58 | 11.16 | 9.03 | 10.63 | 15.00 |

**Table S11**. **Estimated average annual carbon benefit^1^ (million t CO2e) attributable to use of mass timber (CLT and glulam) in projected four-stories-and- higher multifamily residential and nonresidential buildings by U.S. regions under different shared socioeconomic pathways and market adoption scenarios, 2020-2070.**

|  | Multifamily residential | Nonresidential | Multifamily residential + nonresidential |
| --- | --- | --- | --- |

Region --------------------------------------- Low mass timber adoption scenario, average annual value, 2020-2070, million t CO2e --------------------------------------

|  | SSP1 | SSP2 | SSP3 | SSP4 | SSP5 | SSP1 | SSP2 | SSP3 | SSP4 | SSP5 | SSP1 | SSP2 | SSP3 | SSP4 | SSP5 |
| --- | --- | --- | --- | --- | --- | --- | --- | --- | --- | --- | --- | --- | --- | --- | --- |
| Northeast | 0.07 | 0.07 | 0.06 | 0.07 | 0.09 | 0.43 | 0.40 | 0.30 | 0.37 | 0.60 | 0.50 | 0.47 | 0.36 | 0.44 | 0.69 |
| Midwest | 0.08 | 0.08 | 0.07 | 0.08 | 0.10 | 0.72 | 0.69 | 0.55 | 0.66 | 0.97 | 0.80 | 0.77 | 0.62 | 0.73 | 1.07 |
| South | 0.19 | 0.19 | 0.17 | 0.18 | 0.22 | 1.49 | 1.42 | 1.11 | 1.34 | 2.05 | 1.68 | 1.61 | 1.27 | 1.52 | 2.27 |
| West | 0.10 | 0.10 | 0.09 | 0.10 | 0.12 | 0.79 | 0.75 | 0.58 | 0.70 | 1.09 | 0.89 | 0.85 | 0.66 | 0.80 | 1.21 |
| US Total | 0.45 | 0.44 | 0.38 | 0.43 | 0.53 | 3.42 | 3.26 | 2.54 | 3.08 | 4.71 | 3.87 | 3.70 | 2.91 | 3.50 | 5.24 |

------------------------------------ Medium mass timber adoption scenario, average annual value, 2020-2070, million t CO2e ------------------------------------

| Northeast | 0.12 | 0.12 | 0.09 | 0.11 | 0.15 | 0.65 | 0.62 | 0.47 | 0.58 | 0.91 | 0.77 | 0.73 | 0.56 | 0.69 | 1.06 |
| --- | --- | --- | --- | --- | --- | --- | --- | --- | --- | --- | --- | --- | --- | --- | --- |
| Midwest | 0.13 | 0.13 | 0.11 | 0.12 | 0.16 | 1.12 | 1.07 | 0.85 | 1.02 | 1.49 | 1.25 | 1.20 | 0.97 | 1.14 | 1.64 |
| South | 0.31 | 0.30 | 0.27 | 0.29 | 0.35 | 2.29 | 2.19 | 1.72 | 2.07 | 3.11 | 2.60 | 2.49 | 1.99 | 2.37 | 3.47 |
| West | 0.17 | 0.17 | 0.14 | 0.16 | 0.20 | 1.21 | 1.16 | 0.90 | 1.09 | 1.66 | 1.38 | 1.33 | 1.04 | 1.25 | 1.86 |
| US Total | 0.73 | 0.71 | 0.61 | 0.69 | 0.86 | 5.27 | 5.03 | 3.95 | 4.76 | 7.16 | 6.00 | 5.75 | 4.56 | 5.45 | 8.02 |

-------------------------------------- High mass timber adoption scenario, average annual value, 2020-2070, million t CO2e --------------------------------------

| Northeast | 0.16 | 0.15 | 0.12 | 0.15 | 0.20 | 0.83 | 0.79 | 0.60 | 0.74 | 1.15 | 0.99 | 0.94 | 0.72 | 0.89 | 1.35 |
| --- | --- | --- | --- | --- | --- | --- | --- | --- | --- | --- | --- | --- | --- | --- | --- |
| Midwest | 0.18 | 0.17 | 0.15 | 0.16 | 0.21 | 1.43 | 1.37 | 1.10 | 1.31 | 1.89 | 1.61 | 1.54 | 1.25 | 1.47 | 2.10 |
| South | 0.41 | 0.40 | 0.36 | 0.39 | 0.47 | 2.92 | 2.80 | 2.22 | 2.65 | 3.94 | 3.33 | 3.20 | 2.58 | 3.04 | 4.41 |
| West | 0.22 | 0.22 | 0.19 | 0.22 | 0.26 | 1.56 | 1.49 | 1.16 | 1.41 | 2.11 | 1.78 | 1.71 | 1.35 | 1.62 | 2.37 |
| US Total | 0.96 | 0.95 | 0.81 | 0.92 | 1.14 | 6.74 | 6.45 | 5.08 | 6.10 | 9.09 | 7.71 | 7.404 | 5.89 | 7.02 | 10.23 |

-------------------------------------- 100% mass timber adoption scenario, average annual value, 2020-2070, million t CO2e -------------------------------------

| Northeast | 0.25 | 0.25 | 0.20 | 0.24 | 0.32 | 1.27 | 1.21 | 0.92 | 1.13 | 1.72 | 1.52 | 1.46 | 1.12 | 1.37 | 2.04 |
| --- | --- | --- | --- | --- | --- | --- | --- | --- | --- | --- | --- | --- | --- | --- | --- |
| Midwest | 0.59 | 0.57 | 0.50 | 0.55 | 0.69 | 2.21 | 2.12 | 1.72 | 2.03 | 2.87 | 2.80 | 2.69 | 2.22 | 2.58 | 3.56 |
| South | 0.97 | 0.95 | 0.85 | 0.93 | 1.11 | 4.49 | 4.31 | 3.46 | 4.09 | 5.93 | 5.46 | 5.27 | 4.31 | 5.02 | 7.05 |
| West | 0.53 | 0.53 | 0.44 | 0.51 | 0.62 | 2.42 | 2.32 | 1.84 | 2.20 | 3.22 | 2.94 | 2.85 | 2.28 | 2.71 | 3.84 |

| US Total | 2.34 | 2. 30 | 1.99 | 2.23 | 2.75 | 10.38 | 9.96 | 7.94 | 9.45 | 13.74 | 12.73 | 12.26 | 9.93 | 11.68 | 16.49 |
| --- | --- | --- | --- | --- | --- | --- | --- | --- | --- | --- | --- | --- | --- | --- | --- |

^1^ Carbon benefit refers to the sum of (i) avoided emissions due to substitution of mass timber for concrete (difference in embodied fossil GHG emissions between mass timber buildings and functionally equivalent concrete buildings), and (ii) carbon stored in mass timber materials while in use in buildings and in landfills after the buildings are demolished after their useful service.

**Table S12. Projected effects on harvests, inventory, and growth-to-drain ratio by 2070 due to projected new demand for mass timber, assuming U.S. annual forest growth and harvest remain unchanged from 2017 levels (no new demand for mass timber).** Data source: Oswalt et al. [15].

| Scenario |  | Harvests |  | Inventory | |  | Growth-to-drain ratio | |  |
| --- | --- | --- | --- | --- | --- | --- | --- | --- | --- |
|  | (Value in 2070) | Change by 2070 relative to no new  MT demand | | (Value in 2070) | Change by 2070 relative to no new MT  demand | | (Value in 2070) | Change by 2070 relative to no new  MT demand | |
|  | million m^3^ | million m^3^ | % | million m^3^ | million m^3^ | % | million m^3^ | million m^3^ | % |
| No new mass timber adoption scenario | | | | | | | | | |
| No new MT demand | 369.3 | 0.0 | 0.0 | 50,618 | 0.0 | 0.0 | 2.14 | 0.0 | 0.0% |
| Low mass timber adoption scenario | | | | | | | | | |
| SSP1 | 388.6 | 19.3 | 5.2 | 50,250 | -368 | -0.73 | 2.03 | -0.04 | -1.99 |
| SSP2 | 387.4 | 18.2 | 4.9 | 50,265 | -352 | -0.70 | 2.04 | -0.04 | -1.91 |
| SSP3 | 383.2 | 14.0 | 3.8 | 50,340 | -278 | -0.55 | 2.06 | -0.03 | -1.51 |
| SSP4 | 386.2 | 17.0 | 4.6 | 50,284 | -334 | -0.66 | 2.05 | -0.04 | -1.81 |
| SSP5 | 397.0 | 27.8 | 7.5 | 50,121 | -497 | -0.98 | 1.99 | -0.06 | -2.66 |
| Medium mass timber adoption scenario | | | | | | | | | |
| SSP1 | 393.6 | 24.3 | 6.6 | 50,039 | -579 | -1.14 | 2.01 | -0.07 | -3.07 |
| SSP2 | 392.2 | 22.9 | 6.2 | 50,062 | -556 | -1.10 | 2.01 | -0.06 | -2.95 |
| SSP3 | 386.9 | 17.6 | 4.8 | 50,176 | -441 | -0.87 | 2.04 | -0.05 | -2.36 |
| SSP4 | 390.7 | 21.4 | 5.8 | 50,090 | -527 | -1.04 | 2.02 | -0.06 | -2.81 |
| SSP5 | 404.3 | 35.0 | 9.5 | 49,846 | -772 | -1.52 | 1.95 | -0.09 | -4.04 |
| High mass timber adoption scenario | | | | | | | | | |
| SSP1 | 396.9 | 27.7 | 7.5 | 49,868 | -750 | -1.48 | 1.99 | -0.08 | -3.94 |
| SSP2 | 395.3 | 26.0 | 7.1 | 49,897 | -721 | -1.42 | 2.00 | -0.08 | -3.79 |
| SSP3 | 389.3 | 20.0 | 5.4 | 50,042 | -576 | -1.14 | 2.03 | -0.07 | -3.05 |
| SSP4 | 393.6 | 24.3 | 6.6 | 49,933 | -685 | -1.35 | 2.01 | -0.08 | -3.61 |
| SSP5 | 409.0 | 39.8 | 10.8 | 49,626 | -991 | -1.96 | 1.93 | -0.11 | -5.12 |

| 100% mass timber adoption scenario | | | | | | | | | |
| --- | --- | --- | --- | --- | --- | --- | --- | --- | --- |
| SSP1 | 403.8 | 34.5 | 9.4 | 49,365 | -1,252 | -2.47 | 1.96 | -0.14 | -6.38 |
| SSP2 | 401.8 | 32.6 | 8.8 | 49,409 | -1,209 | -2.39 | 1.97 | -0.13 | -6.17 |
| SSP3 | 394.5 | 25.2 | 6.8 | 49,637 | -980 | -1.94 | 2.00 | -0.11 | -5.06 |
| SSP4 | 399.8 | 30.5 | 8.3 | 49,465 | -1,152 | -2.28 | 1.98 | -0.13 | -5.90 |
| SSP5 | 418.6 | 49.3 | 13.4 | 49,000 | -1,618 | -3.20 | 1.89 | -0.17 | -8.09 |

**Table S13. Augmented Dickey-Fuller (ADF) tests and Philips-Perron (PP) tests for unit roots of annual time series on levels of variables used in econometric estimation.**

| Variables | Observations | ADF Stat | ADF Signif. | ADF Lags1 | PP Tau-Stat | PP Signif. | PP Lags | Years Considered |
| --- | --- | --- | --- | --- | --- | --- | --- | --- |
| Real GDP, Northeast | 47 | -0.609 | 0.869 | 0 | -0.330 | 0.872 | 3 | 1975-2021 |
| Real GDP, Midwest | 47 | -0.521 | 0.888 | 0 | -0.530 | 0.886 | 3 | 1975-2021 |
| Real GDP, South | 47 | -0.293 | 0.927 | 1 | 0.364 | 0.980 | 3 | 1975-2021 |
| Real GDP, West | 47 | 0.478 | 0.984 | 1 | 1.286 | 0.997 | 3 | 1975-2021 |
| Prime Rate | 48 | -1.468 | 0.549 | 3 | -1.752 | 0.405 | 3 | 1974-2021 |
| Real Prime Rate | 47 | -2.408 | 0.140 | 0 | -2.466 | 0.124 | 3 | 1974-2021 |
| Consumer Price Index | 47 | -1.249 | 0.652 | 1 | -1.313 | 0.623 | 3 | 1975-2021 |
| Construction Weekly Earnings | 47 | 1.153 | 0.996 | 1 | 2.006 | 0.999 | 3 | 1975-2021 |
| Residential Construction Weekly Earnings | 30 | 0.488 | 0.985 | 0 | 2.323 | 0.999 | 3 | 1996-2021 |
| Nonresidential Construction Weekly Earnings | 30 | 2.223 | 0.999 | 0 | 2.229 | 0.999 | 3 | 1991-2020 |
| Concrete PPI | 47 | 1.036 | 0.995 | 2 | 0.933 | 0.994 | 3 | 1975-2021 |
| Net Immigration Per 1000 Population | 48 | -2.740 | 0.067 | 6 | -1.755 | 0.403 | 3 | 1974-2021 |
| Ln(Real GDP, Northeast) | 47 | -1.610 | 0.480 | 1 | -1.766 | 0.397 | 3 | 1975-2021 |
| Ln(Real GDP, Midwest) | 47 | -1.310 | 0.624 | 0 | -1.238 | 0.657 | 3 | 1975-2021 |
| Ln(Real GDP, South) | 47 | -1.409 | 0.578 | 1 | -1.531 | 0.518 | 3 | 1975-2021 |
| Ln(Real GDP, West) | 47 | -1.132 | 0.702 | 1 | -1.176 | 0.684 | 3 | 1975-2021 |
| Ln(Prime Rate) | 48 | -2.182 | 0.213 | 1 | -1.353 | 0.605 | 3 | 1974-2021 |
| Ln(Consumer Price Index) | 47 | -4.764 | 0.000 | 2 | -7.313 | 0.000 | 3 | 1975-2021 |
| Ln(Construction Weekly Earnings) | 47 | -2.289 | 0.176 | 1 | -3.749 | 0.004 | 3 | 1975-2021 |
| Ln(Residential Construction Weekly Earnings) | 30 | 0.488 | 0.985 | 0 | 0.437 | 0.983 | 3 | 1996-2021 |
| Ln(Nonresidential Construction Weekly  Earnings) | 30 | -0.448 | 0.902 | 0 | -0.457 | 0.900 | 3 | 1991-2020 |
| Ln(Concrete PPI ) | 47 | -2.052 | 0.265 | 3 | -2.918 | 0.043 | 3 | 1975-2021 |
| Ln(Net Immigration per 1000 Population) | 48 | -2.493 | 0.117 | 1 | -1.671 | 0.446 | 3 | 1974-2021 |

**Appendix A**

**Estimating statistical models of the nonresidential construction activities**

*Data and empirical specifications*

We specify two types of reduced-form models (Eqn 3 in the main text) for one measure of new nonresidential construction. The first type, which we identify as “fully specified,” includes potentially endogenous real wages, an inflation-adjusted prime rate of interest, real gross domestic product, the real price of concrete, and geographic indicators intended to account for time-invariant fixed factors related to laws and regulations. The second type of Eqn 3 is more parsimonious, a result of dropping non-statistically significant variables from the first type that can also be projected into the future, while minimizing bias in prediction. The measure of new nonresidential construction is floor area built in the aggregate of new nonresidential units.

Data on both projected variables are highly limited, with only periodic observations of multiyear totals, available over the past four decades. Even accounting for the extra information provided by U.S. Census Bureau Regional data on floor area installed are limited, resulting in limited degrees of freedom for model exploration. The U.S. Census Bureau reports total square footage installed in new nonresidential structures spanning multiple years: 1980-1983, 1984-1986, 1987-1989, 1990-1992, 1993-1995, 1996-1999, 2000-2003, 2004-2007, 2008-2010, 2011-2012, 2013-2018. Data are consistently reported by the U.S. Energy Information Administration [1] major regions: Northeast, Midwest, South, West. Therefore, we have an unevenly temporally based panel. Regressors in the fully specified Eqn 3 and the parsimonious versions of Eqn 3, however, are available at higher frequency and equally temporally spaced. To carry out regressions of versions of Eqn 3, we calculate annual averages of the dependent variable across each of the unevenly distributed time spans and also calculate the time-corresponding annual averages of each of the potential regressors. We then estimate pooled (across regions) versions of Eqn 3 that include region indicators (dummy variables).

To address potential endogeneity of regressors of the main model (Eqn 3), we apply the control function (CF) method [16]. The CF method generates identical parameter estimates as those emanating from two-staged least squares methods in linear models [16] and is capable of managing endogeneity in nonlinear models [17]. The CF method involves a first-stage regression of the potentially endogenous regressor in Eqn 3 on at least one exogenous instrumental variable and the other exogenous regressors in Eqn 3 and introduces the residuals of the first stage regression as an additional regressor in Eqn 3. We identified one potentially endogenous variable in Eqn 3: wage (*W*). Although we do not know the true functional form of Eqn 3, assume that it is linear:

$Q_{i,t}=a_{0}W_{i,t}+\mathbf{b}_{0}'\mathbf{e}_{2i,t}+v_{i,t}$ (A1)

where **e**_2_*_i,t_* = (1, *R_i,t_, Y_i,t_,* ***Z****_i,t_,* ***X****_i,t_*) is a vector of a constant and exogenous variables, *a*_0_ and **b**_0_ are parameters to be estimated, and *v_i,t_* is a residual. If *W_i,t_* were endogenous, then the residuals in an estimate of Eqn A1 would be correlated with *W_i,t_* making the estimate statistically inconsistent.

The CF first-stage regression would be a linear regression of *W_i,t_* on **e**_2_*_i,t_* and one or more additional exogenous regressors contained in **e**_1_*_i,t_*:

$W_{i,t}=\mathbf{c}_{\boldsymbol{1}}'\mathbf{e}_{1i,t}+\mathbf{c}_{\boldsymbol{2}}'\mathbf{e}_{2i,t}+w_{i,t}$ (A2)

from which is produced a vector of predicted residuals:

$\hat{w}_{i,t}=W_{i,t}-({\hat{\mathbf{c}}}_{\boldsymbol{1}}'\mathbf{e}_{1i,t}+{\hat{\mathbf{c}}}_{\boldsymbol{2}}'\mathbf{e}_{2i,t})$ (A3)

where the hats (^) indicate estimates. Finally, Eqn A1 is consistently estimated as:

$Q_{i,t}=aW_{i,t}+\mathbf{b}'\mathbf{e}_{2i,t}+a_{1}\hat{w}_{i,t} +u_{i,t}$ (A4)

because $a_{1}\hat{w}_{i,t}$ controls for the endogenous component in *W_i,t_*.

Recognizing that the regressors in Eqn A4 are available on an annual frequency but the dependent variable *Q* can only be expressed as unevenly spaced period averages, the variables *W* and **e** and the residuals $\hat{w}_{i,t}$ are averaged over the years corresponding to each observation of prior to estimation of Eqn A4. The averaging results in a modified specification:

$\bar{Q}_{i,\tau}=a\bar{W}_{i,\tau}+\mathbf{b}'{\bar{\mathbf{e}}}_{2i,t}+a_{1}{\overline{\hat{w}}}_{i,\tau} +u_{i,\tau}$ (A5)

where the overbars reflect the period-averages and $\tau$ indexes time for the period-averages. Emerging from the regression in Eqn A5 is a consistently estimated set of parameters (*a*, **b**) that can be applied to annual data on the regressors in equally spaced (non-averaged) annual predictions of *Q* out-of-sample. Such a prediction could be useful for generating back-cast estimates of annual nonresidential floor area built and for making scenario-based projections of the future.

Given that we have Census Region data on *Q* and several of the regressors, we specify pooled versions of Eqn A5 that include region dummy indicators. Because we have no a priori notion of the proper functional form of Eqn A5, we estimate four versions: linear, log-linear, and two exponential forms—the Poisson Pseudo-Maximum Likelihood (PPML) and the (Pseudo-) Negative Binomial Maximum Likelihood (NBML) model [18–20].

A potential concern of estimating regressions with uneven period-average data would be unrecognized, and unmodelable, autoregressivity in the dependent variable. Simulations carried out for this study (available from the authors) show that such bias is negligible, particularly if models estimated without recognized autoregressive error structures fit well (e.g., have a high R^2^ in least squares estimation). These simulation results, we contend, alleviate concerns about the effects of potential unmodeled autoregressivity when models estimates are used in projection mode.

Because the dependent variable in these equations was stationary but the right-hand side variables were nonstationary based on augmented Dickey-Fuller and Phillips-Perron tests (the sole exceptions being the natural log-transformed consumer price index and concrete price index), right-hand side variables were expressed in first-differences (S13 Table). To exploit the regional time series variation, we included dummy indicator variables for Census Regions. We tested the goodness of fit of all models by estimating models with an estimation sample and predicted the next two reported quantities of floor area built and identified the best-fitting models based on minimum bias in out-of-sample prediction. To take advantage of the likely difference in predictive power of linear and log-linear specifications, we also evaluated goodness-of-fit out of sample of their equal-weighted ensemble. The equal-weighted ensemble had the best fit out-of-sample in both cases. Poisson pseudo-maximum likelihood (PPML) and negative binomial versions of both models were also evaluated, but models performed poorly out-of-sample and in projection mode and so were not included in the ensembles. Estimates of final models are presented in S2a Table.

*Empirical estimates of nonresidential floor area built models*

Reduced-form control function estimates showed broad significance for all variables. However, tests of parameter estimates were likely over-sized due to low degrees of freedom. Furthermore, some variables, such as endogenous wages, if retained in model specifications for forecasts, would require projections themselves by SSP, complicating projections of both new structures and new floor area. We opted instead to test a few highly parsimonious linear specifications not requiring projections of multiple variables (e.g., [11]). These specifications retained measures of real economic growth (GDP) and interest rates. In these specifications, real interest rates could be broken into nominal rates and inflation, allowing for differential effects of each in the short-run. Additionally, these reduced-form models had indicator variables of Census regions and forced parameter estimates to be identical across regions. Because these models ended up not including potentially endogenous wages, the control function approach was not used.

*Monte Carlo bootstrapped projections of floor area built*

Methods from multiple analysts have, for forecasts, validated ensemble approaches if theory does not guide, or there is uncertainty about, model specification or model functional form [21]. We created two-member ensembles for floor area built, with one member in each a linear specification and the second member a log-linear specification. (Tests of Poisson and negative NBML models showed poor out of sample performance in cross-validation testing on historical data and cases of implausibly large or small predicted values in the Monte Carlo projections). In-sample parameter estimates of the final model estimates for each of the two reduced-form ensemble members are shown in the supplemental data to this article (S2a Table).

In projection mode, we used bootstrapping methods. To carry out this process, the data for the two equation specifications for floor area built shown in S2a Table of this Supplement were bootstrap sampled from historical observations with replacement to generate a bootstrap sample used to estimate parameters for each iteration of a 1,000-iteration Monte Carlo projection of nonresidential floor area built. Projected changes in real GDP were made by sampling from historical annual changes in the natural logarithm of real GDP as a function of a constant and the previous year change in the natural logarithm of real GDP (S2b Table). Projections of real GDP were made for each SSP by starting from the year 2020 and projecting to 2070 using a randomly generated time series, 2020 to 2070, of the change in real GDP but then adjusting the projection so that the projected rate of change in real GDP matched the assumed total GDP change corresponding to each scenario. Projections of the changes in the CPI-adjusted (real) prime rate of interest (S2b Table), used in the first floor area built equation, were specified as a function of a constant and the most recent annual lag of changes in the real prime rate and a random error. In projection mode, the random error was an independent random variable centered at zero with a standard error defined as the model standard error. Projected changes in the natural log of the consumer price index, used in the logarithmic equation estimate for floor area built, were held to zero in the second equation projection of floor area built—that is, consumer price index inflation was held constant at the 2020 level throughout the projection. With projections of annual values of each of the independent variables shown in S2a Table, predicted floor area built in each region (and an exponentiated prediction of ln(floor area built)) were averaged, constituting an ensemble prediction, for each year, 2020-2070.

### Appendix B

**Theoretical Framework of the mass-timber innovation diffusion model**

An important feature of the housing materials market, which plays a critical role in diffusion modeling, is the repeat purchase by the builders. The repeat purchase probability at any point in time can be defined as the revised utility estimate of the product conditional on the first-time usage of the product. In this paper, we adapted a repeat purchase diffusion model developed by Ganguly [22] for the diffusion of structural oriented strand board (OSB) for the US market.

In this model for diffusion of CLT, the utility function, including the base utility of the product $\varphi$ and the additional confidence factor$\alpha$ gained from first-time usage of the product, can be written as $(1-\frac{1}{2}e^{-(\varphi+\alpha)})$may be termed the probability of repeat purchase. Hence, the density function of overall purchases at any point in time should include first-time purchases and repeat purchases.

$f(t)=f_{f}(t)+(1-\frac{1}{2}e^{-(\varphi+\alpha)})F_{f}(t)$ S1

where, $f(t)$ is the probability density function of the purchase of the new generation product with repeat purchase, $(1-\frac{1}{2}e^{-(\varphi+\alpha)})$is the probability of repeat purchase. Here, the initial ‘utility markup’ perception of the product $\varphi$gets adjusted by a ‘confidence factor’ $\alpha$. The value of $\alpha$can be positive or negative depending on whether the market perception of the utility of the product has gone up or down.

Eqn S1 can be written in the following form:

$f(t)=\left[\begin{matrix} -\frac{(1+e^{-(a+bt)})^{-\left(\frac{p}{b}\right)}(1-\frac{1}{2}e^{-\varphi})(e^{-(a+bt)})^{\left(\frac{p}{b}+1\right)}}{(1+e^{-a})^{-\left(\frac{p}{b}\right)}(e^{-a})^{\left(\frac{p}{b}\right)}(1+e^{-(a+bt)})}+\frac{(1+e^{-(a+bt)})^{-\left(\frac{p}{b}\right)}(e^{-(a+bt)})^{\left(\frac{p}{b}\right)}p}{(1+e^{-a})^{-\left(\frac{p}{b}\right)}(e^{-a})^{\left(\frac{p}{b}\right)}} \\ +q\left(1-\frac{(1+e^{-(a+bt)})^{-\left(\frac{p}{b}\right)}(e^{-(a+bt)})^{\left(\frac{p}{b}\right)}}{(1+e^{-a})^{-\left(\frac{p}{b}\right)}(e^{-a})^{\left(\frac{p}{b}\right)}}\right) \end{matrix}\right]$ S2

where,

$(1-\frac{1}{2}e^{-\varphi})$= p (coefficient of trial purchase) S2.1

and

$(1-\frac{1}{2}e^{-(\varphi+\alpha)})$ = q (coefficient of repeat purchase) S2.2

Here, it may be noted that the resultant product usage density function is a ‘degenerate density function,’ as it does not integrate to 1. The degeneracy of the density function is by choice, as it reflects the actual market situation where the sales of the product, which has appealing features and competitive advantage over its competitor(s), do not die down automatically.

Accordingly, the parameters that would be modified to develop different mass timber adoption and diffusion scenarios are as follows:

- *Coefficient of trial purchase (p):* the coefficient of trial purchase is solely a function of the utility markup of CLT (*ϕ*)as can be seen in Eqn S2.1. Hence the trial purchase will be

dependent on the perceived utility obtained from the new generation product or system, in this case, mass timber structural system (CLT and glulam), as compared to the traditional system.


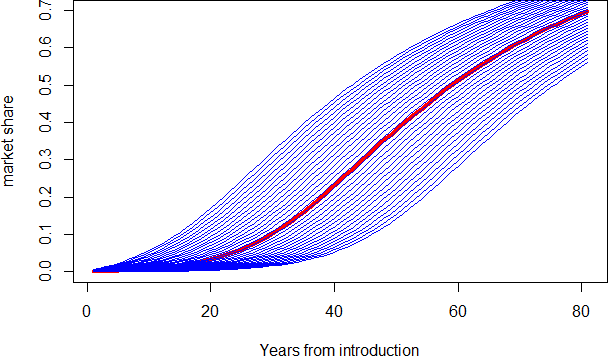

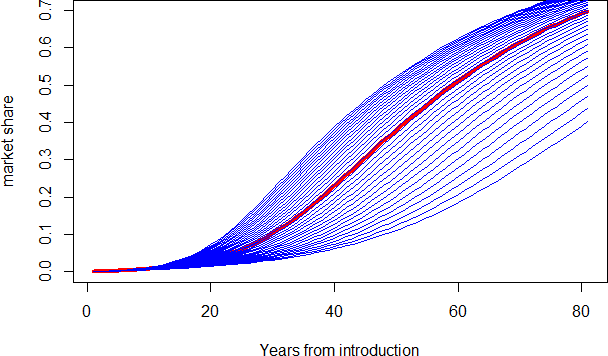


b = 0.5

a = 1.5

a = 0.5

b = 1.5

- *Coefficient of repeat purchase (q):* the perceived utility of the product or system gets revised after a firms’ trial use of the product, as denoted by the confidence factor (α). This gets factored in the coefficient of repeat purchase as is denoted in Eqn S2.2.
- Finally, the two parameters that can vary significantly by country and by industry are (i) how informed the market is, i.e., the *coefficient of lack of base information level (a)*, and (ii) the *coefficient of the rate of information dissemination (b)*. These coefficients are not only dependent on countries but can get heavily influenced by industry outreach, media coverage, and government support schemes.

We adopted the parameter developed by Ganguly [22] for structural OSB in the U.S. construction sector as baseline parameters. These baseline parameters are presented in Table A1. Then we factored in variations to that parameter based on the baseline. To be able to develop adoption diffusion scenarios for U.S., a sensitivity analysis was undertaken on each of the parameters’ baselines. It may also be noted that all the parameters influence the diffusion curve differently. In Figure A1, the red line represents the curve based on the parameters in Table A1. When the parameters ‘a’ and ‘b’ are increased and decreased by 50%, the diffusion curves change differently, as shown in Figure A1. When the value of ‘a’ lack of base information is decreased by 50%, the diffusion curve shifts up early on in the diffusion trajectory.

Table A1: Estimated starting parameter for the diffusion model, as per Ganguly [22].

| Coefficient | Definition of the Coefficient | Estimated Parameters |
| --- | --- | --- |
| *a* | Coefficient of lack of base information | 4.399 |
| *b* | Coefficient of information dissemination | 0.1305 |
| *p* | Coefficient of trial purchase | 0.295 |
| *q* | Coefficient of repeat purchase | 0.9211 |

Figure A1: Parameter variation and diffusion curve

However, when the parameter ‘b’ coefficient of information dissemination is increased by 50%, there is a delay in the shift of the diffusion curve, as information dissemination can follow trial adoption, and hence there is a lag. Similarly, the changes in parameters ‘p’ and ‘q’ would impact the curve differently. In this paper, we develop U.S.-specific innovation diffusion by varying these four parameters.

### SI References

1. U.S. Energy Information Administration. Commercial Buildings Energy Consumption Survey (CBECS). 2023 [cited 12 Nov 2022]. Available: https://www.eia.gov/consumption/commercial/data/2018/bc/xls/b10.xlsx; accessed June 174, 2023; accessed November 12, 2022.

2. USDC-Bureau of Economic Analysis. Regional Data: GDP and Personal Income. SAGDP9S Real GDP by State: All Industry Total (Millions of chained 1997 dollars). 2022 [cited 6 Mar 2022]. Available: https://apps.bea.gov/iTable/iTable.cfm?reqid=70&step=1&acrdn=2; accessed March 06, 2022

3. Federal Reserve of St. Louis (FRSL). Bank Prime Loan Rate, Percent, Monthly, Not Seasonally Adjusted. 2021 [cited 15 Dec 2021]. Available: https://fred.stlouisfed.org/series/MPRIME; accessed December 15, 2021

4. USDC-Bureau of Labor Statistics (USDC-BLS)a. CPI for All Urban Consumers (CPI-U). 2021 [cited 21 Nov 2021]. Available: https://www.bls.gov/cpi/data.htm; accessed November 21, 2021.

5. USDC-Bureau of Labor Statistics (USDC-BLS)b. Super Sector: Construction, Industry: Construction. Average Weekly Earnings Of Production And Nonsupervisory Employees. 2021 [cited 15 Dec 2021]. Available: http://www.bls.gov/data/; accessed December 15, 2021.

6. USDC-Bureau of Labor Statistics (USDC-BLS)c. Super Sector: Construction, Industry: Residential Building. Average Weekly Earnings Of Production And Nonsupervisory Employees. 2021 [cited 15 Dec 2021]. Available: http://www.bls.gov/data/; accessed December 15, 2023.

7. USDC-Bureau of Labor Statistics (USDC-BLS)d. Super Sector: Construction, Industry: Nonresidential Building. Average Weekly Earnings Of Production And Nonsupervisory Employees. 2021 [cited 15 Dec 2021]. Available: http://www.bls.gov/data/; accessed December 15, 2021.

8. USDC-Bureau of Labor Statistics (USDC-BLS)e. Producer Price Indexes, PPI Databases, Industry Data. 2021 [cited 15 Dec 2021]. Available: https://www.bls.gov/ppi/databases/; accessed December 15, 2021.

9. Macrotrends. U.S. Net Migration Rate 1950-2023. 2023 [cited 6 Mar 2023]. Available: https://www.macrotrends.net/countries/USA/united-states/net-migration; accessed March 06, 2023

10. U.S. Census Bureau. New residential construction, historical data. 2022 [cited 20 Jul 2022]. Available: https://www.census.gov/construction/nrc/xls/quar_co_purpose_cust.xls; accessed July 20, 2022.

11. Prestemon JP, Nepal P, Sahoo K. Housing starts and the associated wood products carbon storage by county by Shared Socioeconomic Pathway in the United States. PLoS One. 2022;17: e0270025. doi:https://doi.org/10.1371/journal.pone.0270025.

12. U.S. Census Bureau. American Housing Survey (AHS) Table Creator. 2022 [cited 17 Jul 2022]. Available: https://www.census.gov/programs-surveys/ahs/data/interactive/ahstablecreator.html?s_areas=00000&s_year=2019&s_tablename=TABLE1&s_bygroup1=1&s_bygroup2=1&s_filtergroup1=1&s_filtergroup2=1; accessed July 17, 2022.

13. Dolan J, Wilson A, Brandt K, Bender D, Wolcott M. Structural design process for estimating cross-laminated timber use factors for buildings. BioResources. 2019;14: 7247–7265.

14. Puettmann M, Pierobon F, Ganguly I, Gu H, Chen C, Liang S, et al. Comparative LCAs of conventional and mass timber buildings in regions with potential for mass timber penetration. Sustainability. 2021;13. doi:10.3390/su132413987

15. Oswalt SN, Smith WB, Miles PD, Pugh SA. Forest Resources of the United States, 2017: a technical document supporting the Forest Service 2020 RPA Assessment. USDA Forest Service Gen. Tech. Rep. WO-97. Washington DC; 2019.

16. Hausman J. Specification tests in econometrics. Econometrica. 1978;46: 1251–1271.

17. Wooldridge J.M. Control function methods in applied econometrics. J Hum Resour. 2015;50: 420–445.

18. Gourieroux C, Monfort A, Trognon A. Pseudo maximum likelihood methods: applications to Poisson models. Econometrica. 1984;52: 701–720.

19. Santos Silva JMC, Tenreyro S. The log of gravity. Rev Econ Stat. 2006;88: 641–658. doi:doi.org/10.1162/rest.88.4.641

20. Santos Silva JMC, Tenreyro S. On the existence of the maximum likelihood estimates in Poisson regression. Econ Lett. 2010;107: 310-312.

21. Armstrong JS. Combining forecasts. In: Armstrong JS, editor. Principles of Forecasting: A Handbook for Researchers and Practitioners. Norwell, MA: Armstrong, J.S.; 2001. pp. 1–19.

22. Ganguly I. Modeling Alternatives and Visualization of Product Adoption and Usage in the Residential Construction Industry. University of Washington, Seattle, WA. 2008.
